# Cyclophilin D‐dependent mitochondrial permeability transition amplifies inflammatory reprogramming in endotoxemia

**DOI:** 10.1002/2211-5463.13091

**Published:** 2021-02-13

**Authors:** Balazs Veres, Krisztian Eros, Csenge Antus, Nikoletta Kalman, Fruzsina Fonai, Peter Balazs Jakus, Eva Boros, Zoltan Hegedus, Istvan Nagy, Laszlo Tretter, Ferenc Gallyas, Balazs Sumegi

**Affiliations:** ^1^ Department of Biochemistry and Medical Chemistry Medical School University of Pecs Hungary; ^2^ MTA‐PTE Nuclear‐Mitochondrial Interactions Research Group Pecs Hungary; ^3^ Szentagothai Janos Research Center University of Pecs Hungary; ^4^ Institute of Biochemistry Biological Research Centre Szeged Hungary; ^5^ Institute of Biophysics Biological Research Centre Szeged Hungary; ^6^ SeqOmics Biotechnology Ltd Morahalom Hungary; ^7^ Department of Medical Biochemistry Semmelweis University Budapest Hungary

**Keywords:** antioxidant, cyclophilin D, endotoxin, gene expression, inflammation, liver, mitochondria, oxidative stress, reprogramming, Toll‐like receptor

## Abstract

Microorganisms or LPS (lipopolysaccharide), an outer membrane component of Gram‐negative bacteria, can induce a systemic inflammatory response that leads to sepsis, multiple organ dysfunction, and mortality. Here, we investigated the role of cyclophilin D (CypD)‐dependent mitochondrial permeability transition (mPT) in the immunosuppressive phase of LPS‐induced endotoxic shock. The liver plays an important role in immunity and organ dysfunction; therefore, we used liver RNA sequencing (RNA‐seq) data, Ingenuity^®^ Pathway Analysis (IPA ^®^) to investigate the complex role of mPT formation in inflammatory reprogramming and disease progression. LPS induced significant changes in the expression of 2844 genes, affecting 179 pathways related to mitochondrial dysfunction, defective oxidative phosphorylation, nitric oxide (NO) and reactive oxygen species (ROS) accumulation, nuclear factor, erythroid 2 like 2 (Nrf2), Toll‐like receptors (TLRs), and tumor necrosis factor α receptor (TNFR)‐mediated processes in wild‐type mice. The disruption of CypD reduced LPS‐induced alterations in gene expression and pathways involving TNFRs and TLRs, in addition to improving survival and attenuating oxidative liver damage and the related NO‐ and ROS‐producing pathways. CypD deficiency diminished the suppressive effect of LPS on mitochondrial function, nuclear‐ and mitochondrial‐encoded genes, and mitochondrial DNA (mtDNA) quantity, which could be critical in improving survival. Our data propose that CypD‐dependent mPT is an amplifier in inflammatory reprogramming and promotes disease progression. The mortality in human sepsis and shock is associated with mitochondrial dysfunction. Prevention of mPT by CypD disruption reduces inflammatory reprogramming, mitochondrial dysfunction, and lethality; therefore, CypD can be a novel drug target in endotoxic shock and related inflammatory diseases.

## Graphical abstract

Disruption of CypD prevents mPT and attenuates LPS‐induced complex changes, resulting in the maintenance of stable mitochondrial structure and reduced ROS‐related signaling. The effects of CypD disruption were reduction of LPS‐induced reprogramming and protection of mitochondria and OXPHOS complexes, which have an important effect on mouse survival. CypD‐dependent mPT inhibition may be a novel drug target for inflammatory diseases.

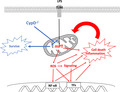

**Abbreviations**
4‐HNE: 4‐hydroxynonenal
adjPval: false discovery rate adjusted p‐values by Benjamini–Hochberg methodology
Akt2: Akt serine/threonine kinase 2
Atf4: activating transcription factor 4
Cat: catalase
c‐Jun: jun proto‐oncogene, AP‐1 transcription factor subunit
c‐Myc: v‐myc avian myelocytomatosis viral oncogene homolog
Cox7c: cytochrome C oxidase subunit 7C
Csf1: colony stimulating factor 1
Cxcl2: C‐X‐C motif chemokine ligand 2
CypD/Ppif: cyclophilin D/Peptidyl‐prolyl cis‐trans isomerase F
DAMPs: damage‐associated molecular patterns
DEG: differentially expressed gene
ePCR: emulsion polymerase chain reaction
Erk1/2: extracellular signal‐regulated kinase ½
Fas: fas cell surface death receptor
FDR: false discovery rate
Gapdh: glyceraldehyde‐3‐phosphate dehydrogenase
Gapdh: glyceraldehyde‐3‐phosphate dehydrogenase
Gpx1: glutathione peroxidase
Gsr: glutathione S‐reductase
Gstt: glutathione S‐transferase θ 1
Hif1a: hypoxia‐inducible factor 1α
Hnf4a: hepatocyte nuclear factor 4 alpha
Ifnγ: interferon γ
iNOS/Nos2: inducible NO synthase
IPA®: Ingenuity® Pathway Analysis
IPKB: Ingenuity® Pathway Knowledge Base
Irak3: interleukin‐1 receptor‐associated kinase
Lbp: lipopolysaccharide binding protein
LPS: lipopolysaccharide
Lrpprc: leucine‐rich pentatricopeptide repeat containing
MAPK: mitogen‐activated protein kinase
mito12SrRNA: mitochondrial 12S rRNA
mPT: mitochondrial permeability transition
Mrpl: mitochondrial ribosomal protein L
mtDNA: mitochondrial DNA
mtND5: mitochondrial NADH dehydrogenase subunit 5
Myd88: myeloid differentiation primary response 88
Ndufb5: NADH:ubiquinone oxidoreductase subunit B5
NF‐ κB: nuclear factor κB
Nfkbia: inhibitor of nuclear factor κB kinase subunit α
Nfkbib: inhibitor of nuclear factor κB kinase subunit β
Nik: NF‐κβ‐inducing kinase
Nlrp12: NLR family pyrin domain‐containing 12
NO: nitric oxide
NRF2/Nfe2l2: nuclear respiratory factor 2/nuclear factor, erythroid 2 like 2
Prdx1: peroxiredoxin 1
qPCR: quantitative polymerase chain reaction
Rela: RELA proto‐oncogene, NF‐κB subunit
RIN: RNA integrity number
RNA‐seq: whole transcriptome mRNA sequencing
ROS: reactive oxygen species
Sdhc: succinate dehydrogenase complex subunit C
Sigirr: single Ig and TIR domain containing
Sod2: superoxide dismutase 2
SSS: DNA standard stock solution;
Tab2: TGF‐β Activated Kinase 1 Binding Protein 2
Tank: TRAF family member‐associated nuclear factor‐κB activator
Tbk: Tank‐binding kinase
TLR: toll‐like receptor
Tnfaip3/A20: TNF alpha induced protein 3
TNFR: tumor necrosis factor α receptor
Tnfrsf1a: TNF receptor superfamily member 1A
Tnfrsf1b: TNF receptor superfamily member 1B
TNFα: tumor necrosis factor α
WT: wild type
β2m: β2 microtubulin

## Introduction

Microorganisms or LPS, an outer membrane component of Gram‐negative bacteria, can induce a systemic inflammatory response that leads to sepsis, multiple organ dysfunction, and mortality [1]. In the early phase of sepsis, a hyperinflammatory state exists, during which a large quantity of cytokines and chemokines are produced, leading to redox imbalance followed by innate immune dysfunction and adaptive immune suppression that eventually results in multiple organ failure and death [1, 2]. With improvements in intensive care management, the rate of early sepsis mortality has been reduced; therefore, the search for sepsis‐induced alterations in immune function and cellular damage has been concentrated on the late phase [2]. The importance of elucidating novel therapeutic targets, affecting the late phase of sepsis‐induced alterations in immune function and cellular damage, is highlighted by the very limited success of anti‐inflammatory therapies to date [1]. Animal studies raised the possibility that activation of the late‐phase inflammatory response could be advantageous [3]. The exact molecular mechanism of late‐phase multiple organ dysfunction and mortality in septic shock is not well characterized yet; however, it has been suggested that mitochondrial damage can play a role [4, 5].

In the liver, microorganism‐ or LPS‐induced inflammation can contribute to the death of hepatocytes, releasing damage‐associated molecular patterns (DAMPs), including those originating from the mitochondria, which process can contribute to disease progression [6, 7, 8]. Therefore, prevention of the extensive, predominantly necrotic cell death would likely decrease disease progression and promotes survival during LPS‐induced shock. It has been shown that oxidative stress is a significant contributor to LPS‐induced inflammatory signaling and fatality, which can be attenuated by antioxidants or NADPH oxidase 2 disruption [9, 10]. Unfortunately, antioxidants are effective only at millimolar concentrations and, thus, are not applicable to human studies. It has been reported that the opening of mPT, a nonspecific channel in the inner membranes of mitochondria, induces superoxide flashes [11] and propagates necrotic cell death [12, 13] and in this way contributes to the release of a large quantity of mitochondrial proteins including DAMPs [14]. These signal molecules, via TLR activation, maintain the vicious inflammatory cycle propagating organ dysfunction. The disruption of the peptidyl‐prolyl cis‐trans isomerase F (Ppif) gene product, CypD, an important regulator of the mitochondrial permeability transition, blocks the elevated Ca^++^ induced mPT and reduces ROS production and cell death [12, 13]. A nonspecific CypD inhibitor has been demonstrated to reduce the inflammatory response [15]. Our previous data showed that disruption of CypD protected against the LPS‐induced development of acute lung injury [16], inhibited LPS‐induced mPT pore opening in peritoneal macrophages, and reduced the LPS‐induced inflammatory response, mitochondrial depolarization, and cellular and mitochondrial ROS production [16, 17], suggesting that CypD is an interesting novel molecular target for the treatment of inflammatory diseases. Hepatocytes play a central role in the acute‐phase response, supplying circulation of different types of immune‐active proteins during stress conditions, including sepsis [18]. The liver also exerts an integrative role in metabolism, the expression of inflammatory proteins, and the regulation of the complement system [19], therefore, playing a critical role in the initiation of multiple organ failure in sepsis.

Microorganisms or LPS has been reported to initiate reprogramming of gene expression in cells and animal models, which contributes to the development of a hyperinflammatory state, and later an immunosuppressive phase [20, 21]. Most studies have preponderantly focused on the active inflammatory state of macrophages [22, 23], with only a few addressing inflammatory reprogramming in the liver [20, 22]. Sepsis‐induced fatality occurs predominantly during the late phase; therefore, in the present study, we strived to understand the molecular mechanisms of inflammatory reprogramming during the immunosuppressive phase of sepsis and the role of CypD‐dependent mPT in these processes.

## Results

### Effect of CypD deficiency on gene expression patterns in LPS‐induced endotoxic shock

**Fig. 1:**
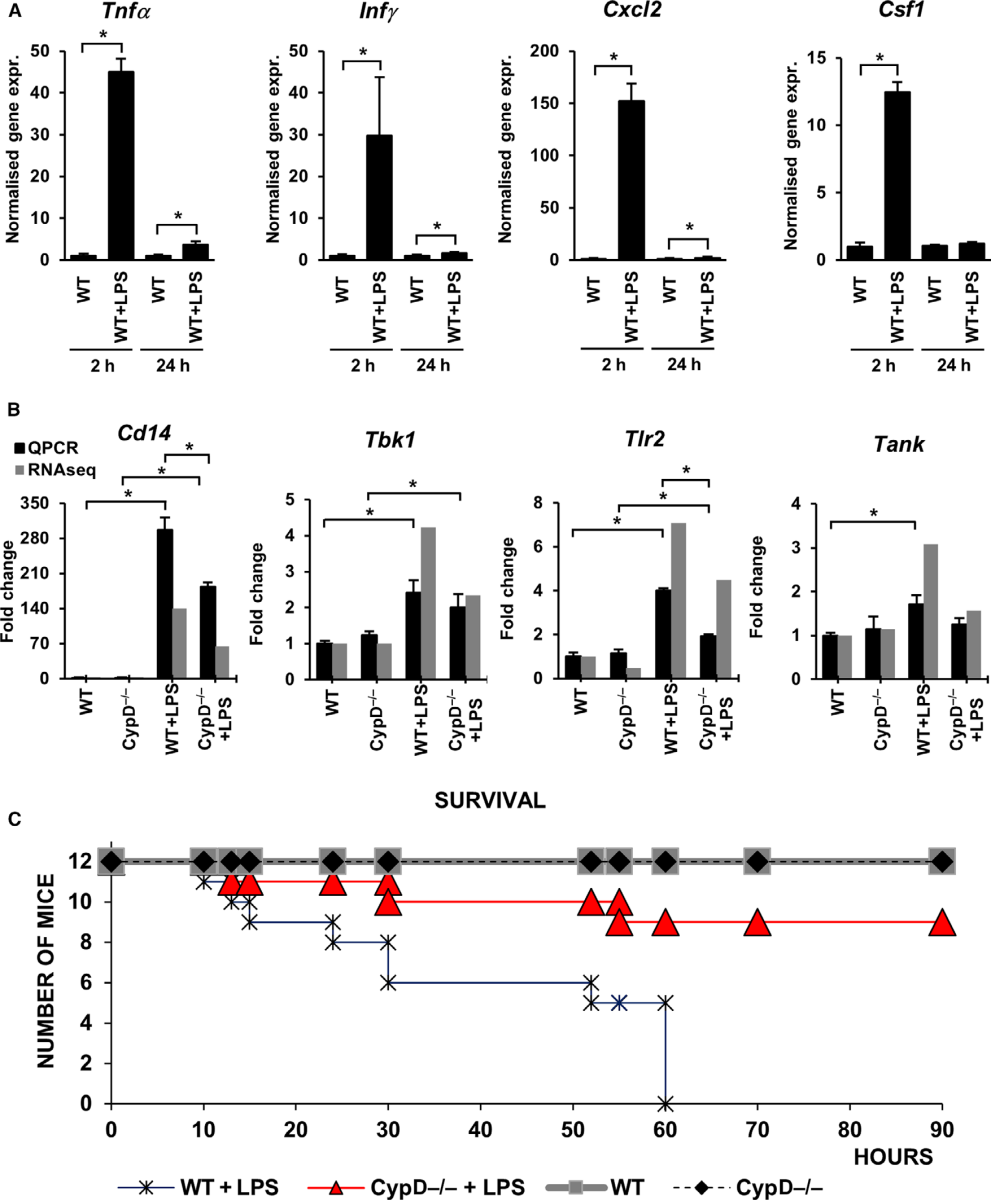
Cyclophilin D disruption alters the inflammatory response during LPS‐induced sterile shock in mouse liver. (A) LPS‐induced expression of Tnfα, Ifnγ, Cxcl2, and Csf1 at 2 h (proinflammatory phase) and 24 h (immunosuppressed phase) were determined by qPCR. Expression values were normalized to β‐actin. Data are presented as mean ± SEM of FC values (*n* = 5) relative to the expression in the WT group. *P < 0.05, independent samples *t*‐test at both time points. (B) qPCR validation of *Cd14*, *Tbk1, Tlr2,* and *Tank* mRNAs levels in sequencing data during LPS‐induced endotoxemia in WT and CypD^−/−^ mice liver. Data are presented as mean ± SEM of FC values (*n* = 5) normalized to the expression in WT mice. *P < 0.05, one‐way ANOVA followed by LSD *post hoc* comparisons on qPCR data. (C) Effect of LPS exposure on the survival of WT and CypD^−/−^ mice (*n* = 12, labeling in the Figure).

Wild‐type (WT) and CypD^−/−^ mice were treated with 40 mg·kg^−1^ LPS and sacrificed after 2 or 24 h, and the liver was removed and processed for RNA isolation. The inflammatory state of mice was characterized by the expression of tumor necrosis factor‐α (*Tnfα*), C‐X‐C motif chemokine ligand 2 (*Cxcl2*), colony‐stimulating factor 1 (*Csf1*), and interferon‐γ (*Ifnγ*) (Fig. 1A), demonstrating an inflammatory burst at 2 h and an immunosuppressive phase at 24 h. At that late phase, LPS administration induced elevated expression of the microbial infection‐related Toll‐like receptor 2 (*Tlr2*), *Cd14*, TANK‐binding kinase 1 (*Tbk1*), and TRAF family member‐associated nuclear factor‐κB activator (*Tank*) and this upregulation was profoundly attenuated in LPS‐stressed CypD^−/−^ animals (Fig. 1B).

Survival data reveal that in WT mice, mortality occurred 10 h after LPS administration and there was a profound increase during the immunosuppressive phase, while CypD deficiency aided the survival of endotoxemic mice (Fig. 1C). Therefore, the LPS‐induced reprogramming of gene expression was analyzed during the immunosuppressive phase at 24 h using mRNA sequencing of the entire transcriptome in liver samples.

**Fig. 2:**
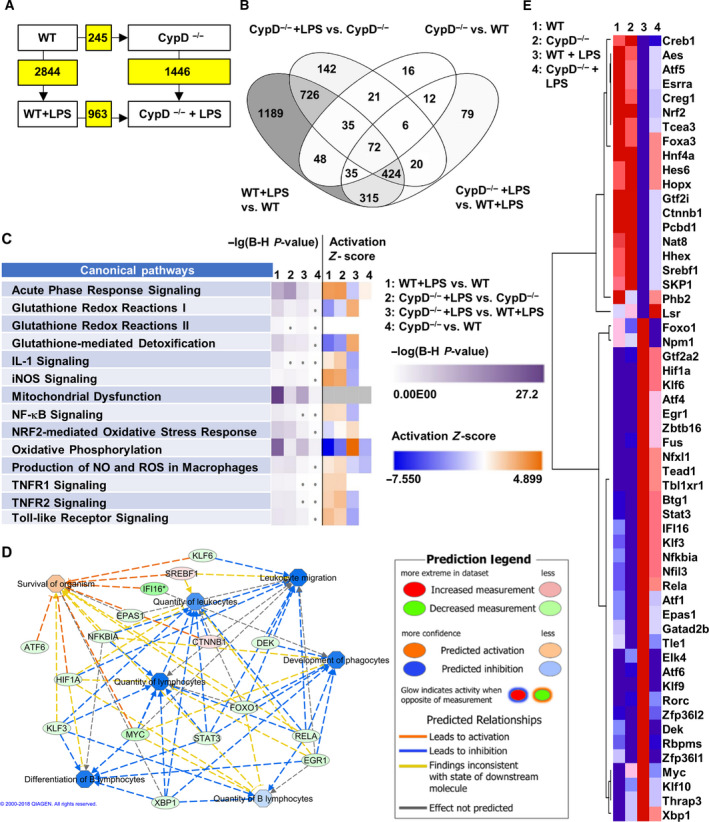
Cyclophilin D disruption alters gene expression reprogramming following LPS exposure in mouse liver. (A) Number of DEGs among the given comparisons. Kal’s *Z*‐test, adjPval < 0.05, [FC] > 1.5. (B) Venn diagram demonstrates proportions of DEGs among each of the comparisons (*n* = 5). (C) Comparing the LPS‐induced changes in WT and CypD^−/−^ mice for the pathways we analyze in detail in this work. Purple shades represent –lg of adjPval for the enrichment in the given pathway. Orange and blue shades represent predicted activation or suppression of the given pathway, respectively, by Z‐scores calculated based on the expressional changes (*n* = 5). (D) Modeling the effect of a subset of differentially expressed transcription factors in LPS‐stressed groups on immune cell activation and organismal survival. Legend is provided in the figure. (E) Heat map representing the expression tendencies of transcription factors differentially induced in expression by LPS in WT and CypD^−/−^ mouse livers by at least a 1.5‐fold expression level change and adjPval. < 0.05 (*n* = 5).

Figure S2 A shows the read alignment of the *Ppif* gene in WT and CypD^−/−^ mice, indicating the deletion of exons 2, 3, and 4. Visualizing the expression profiles of DEGs among the experimental groups shows large‐scale differences in LPS‐induced alterations between WT and CypD^−/−^ mice (Fig. S1A). The Venn diagram demonstrates the proportions of DEGs among the groups (Fig. 2B). A total of 3140 differentially expressed genes were found, 726 of which were identical in the LPS‐treated WT and CypD^−/−^ mice, while an additional 72 genes occurred in all comparisons. LPS exposure induced the differential expression of 1189 and 142 genes unique to WT and CypD^−/−^ background, respectively. CypD deficiency in itself induced the differential expression of altogether 245 genes. Interestingly, among these only 16 genes were unique to this comparison and were not induced by LPS exposure.

A total of 2715 and 1369 individual genes were mapped to the Ingenuity^®^ Pathway Knowledge Base (IPKB), demonstrated significantly different expression following LPS administration in WT and CypD^−/−^ mice, respectively (Fig. 2A). Of these genes, 881 (32.4%) and 426 (31.1%) were upregulated, while 1834 (67.6%) and 943 (68.9%) were suppressed in WT and CypD^−/−^ mice, respectively. These data indicate that the number of LPS‐affected genes in WT mice was halved in CypD^−/−^ mice, while there was a uniform 2‐fold increase in downregulation compared with upregulation in the immunosuppressive phase of endotoxemia, irrespective of the presence or absence of CypD.

Using a 0.05 cutoff for the ‐lg of FDR adjusted p‐values by Benjamini–Hochberg method (adjPval > 1.3), Ingenuity® Pathway Analysis revealed DEGs to be significantly enriched in 179 pathways in the LPS‐treated WT mice (Table S1), while in CypD^−/−^ mice, LPS induced alterations in 165 pathways (Table S2). Figure 2C summarizes in more detail the effects of CypD deficiency in case of LPS exposure on the pathways discussed. Twenty‐four hours after LPS administration, the analysis revealed persistent activation of pathways related to microbial exposure, such as TLR, TNFR1, and TNFR2, nuclear factor κB (NF‐κB), inducible NO synthase (iNOS/*Nos2*) signaling, and acute‐phase response. Conversely, cellular functions and defense mechanisms in WT mice were found still to be compromised due to LPS exposure, as revealed by the significant enrichment of DEGs in the gene sets related to mitochondrial dysfunction, oxidative phosphorylation, and Nrf2‐mediated oxidative stress response pathways (Fig. 2C, Table S1). Disruption of CypD, and thus the prevention of mPT formation, partially alleviated these processes via attenuation of inflammatory signaling and preservation of cellular functions (Fig. 2C, Table S2). Most importantly, CypD disruption, when compared with the WT background, affected the processes of acute‐phase signaling by expressional changes in opposite directions to the LPS‐induced alterations in both WT and CypD^−/−^ animals.

The expression levels of 55 transcription factors changed significantly following LPS treatment in WT mice as compared with CypD^−/−^ mice (Fig. 2E), which may serve as a molecular basis for the dramatic differences in LPS‐induced gene expression. A subset of these transcription factors is selected based on IPKB annotations related to immune cell activation and organismal survival, and their connections are visualized in Fig. 2D. Modeling the effect of their differential expression on these functions suggests attenuated immune cell activation and this way an effect on survival after LPS exposure under CypD deficiency. To demonstrate the differential expression of hypoxia‐inducible factor 1α (*Hif1α)*, RELA proto‐oncogene, NF‐κB subunit (*Rela)*, and hepatocyte nuclear factor 4 α (*Hnf4 α)* transcription factors among the groups, the read alignment is visualized in Fig. S2B.

### Regulation of Toll‐like receptor signaling by disruption of CypD in endotoxic shock

**Fig. 3:**
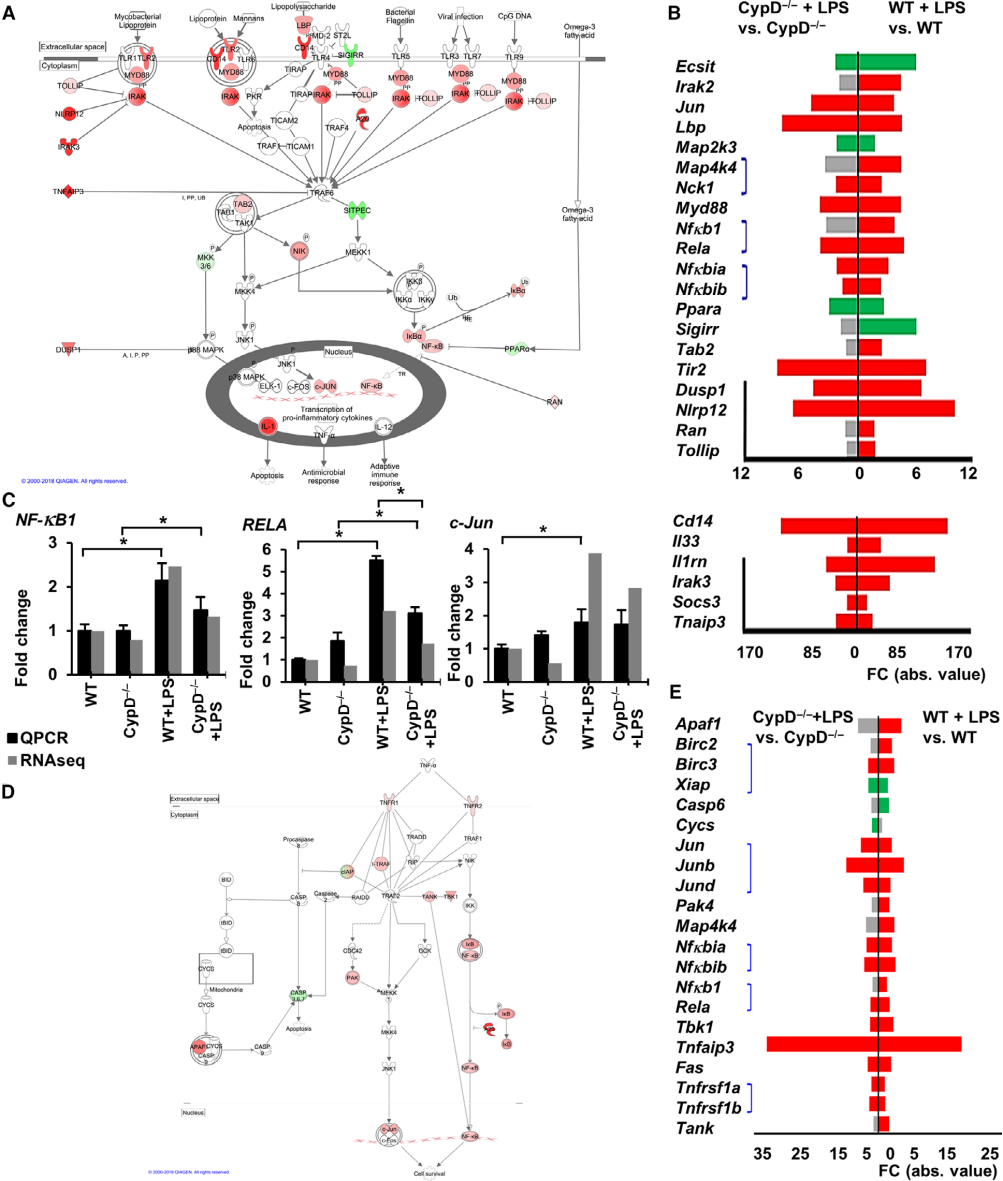
Canonical pathways of Toll‐like receptor, TNFR1 and TNFR2 signaling in LPS‐induced sterile shock model. (A) Visualizing LPS‐induced DEGs in the Toll‐like receptors pathway in WT mice liver. Red signs show upregulated genes while green signs represent downregulated genes. Genes with no significant changes labeled with white background. On the left side of the figure highly expressed inhibitory genes demonstrating immunosuppressive stage (modified). (B) Demonstrating the effect of CypD^−/−^ in case of LPS exposure on DEGs in the TLR pathway. Red bars show significantly upregulated gene, while green bars represent downregulated genes and gray bars show genes not significantly affected. Kal’s Z‐test, adjPval < 0.05, [FC] > 1.5. Black line on the left marks the inhibitory genes in this pathway. Blue lines connect the members of the same multimolecular complexes (in order, from top to bottom: NIK compl., NF‐κB compl., I‐κB compl.). Please notice, genes with high FC values are presented on a different scale (*n* = 5). (C) qPCR validation of RNA‐Seq data for *NFkB1, Rela* and *c‐Jun* expression. Data are presented as mean ± SEM of FC values normalized to the expression in WT mice, (*n* = 5). *P < 0.05, one‐way ANOVA followed by LSD *post hoc* comparisons on qPCR data. (D) Visualizing LPS‐induced DEGs in TNFR1 and TNFR2 pathways in WT mice (combined). Red signs show upregulated genes while green signs represent downregulated genes. Genes with no significant changes labeled with white background. (E) Demonstrating the effect of CypD disruption on the DEGs in TNFR1 and TNFR2 pathways, (*n* = 5). Red bars show significantly upregulated gene while green bars represent downregulated genes, gray bars show genes not significantly affected. Kal’s Z‐test, adjPval < 0.05, [FC] > 1.5. Blue lines connect the members of the same multimolecular complexes (in order, from top to bottom: cIAP compl., Jun compl., I‐κB compl, NF‐κB compl., TNFR compl)

During the late phase of endotoxemia in WT mice, the TLR pathway was still affected by LPS administration in hepatic tissue (adjPval = 3.81E + 00, ratio = 23/76) as revealed by the high expression of adapter proteins mediating signal transmission, such as lipopolysaccharide binding protein (*Lbp*), CD14, and myeloid differentiation primary response 88 (*Myd88*) (Fig. 3A,B). Moreover, we observed elevated expression of members of the TLR2 complex and suppressed transcription of the negative modulator, single Ig, and TIR domain‐containing (*Sigirr*), the protein products of which are located at the plasma membrane. Twenty‐four hours after LPS exposure, the expression levels of key signaling molecules such as members of the interleukin‐1 receptor‐associated kinase 3 (*Irak3*), TGF‐β‐activated kinase 1 binding protein (*Tab2*), and NF‐κB‐inducing kinase (NIK) complexes were still elevated, leading to transcriptional activation of NF‐κB, Jun proto‐oncogene, AP‐1 transcription factor subunit (c‐Jun) complexes, and maintenance of immune cell activation and inflammation. These processes were also supported by the increased expression of *NF‐κB1*, *Rela,* and c‐Jun in WT mice following LPS exposure (Fig. 3A,B) and validated by qPCR (Fig. 3C). However, in our study some of the negative regulators of pathogen sensing signaling cascade were altered in expression 24 hours after LPS exposure, marked in Fig. 3A,B.

According to the results of pathway analysis, LPS‐induced activation of NF‐κB signaling (adjPval = 2.68E + 00, ratio = 38/181) and TLR activation was less impacted in the CypD^−/−^ mice compared with WT mice (adjPval = 1.82E + 00, ratio = 21/181) and (adjPval = 3.39E + 00, ratio = 15/76), respectively. Contrary to the observation on WT animals (adjPval = 2.25E + 00, ratio = 22/92), enrichment in the IL‐1 signaling pathway was nonsignificant (adjPval = 6.24E‐01, ratio = 9/92) (Fig. 2C, Table S2). Following LPS exposure, CypD deficiency attenuated signal transduction in the TLR pathway at the transcriptional level (Fig. S1B). Ten genes showed differential expression when comparing the LPS‐exposed groups. To further demonstrate the differential expression of genes involved in TLR signaling, read alignments of *Irak3, Cd14, Tab2,* and *Tlr2* are visualized in Fig. S2 C.

### TNFR signaling

Hepatocytes produced a very small amount of TNFα during the late stage of endotoxemia (Fig. 1A), but TNFα can be released by the cellular elements of blood and reach the cell surface of hepatocytes [18]. Analysis of the TNFR pathways in WT mice revealed TNFR1 (adjPval = 2.06E + 00, ratio = 14/50) and TNFR2 signaling (adjPval = 3.11E + 00, ratio = 12/30) pathways to be still significantly impacted by LPS exposure (Fig. 2C, Table S1). After 24 hours, the expression levels of TNFR superfamily member 1A (*Tnfrsf1a*) and Fas cell surface death receptor (*Fas*) were found to be slightly elevated, which may propagate TNFα‐induced cytotoxicity and inflammation. Moreover, LPS increased the expression of TNFR superfamily member 1B (*Tnfrsf1b*), which has anti‐apoptotic and antioxidant effects [24] (Fig. 3D, E, S1 C). Downstream of TNFRs, the expression levels of certain complexes that participate in NF‐κB and c‐Jun activation were still elevated, accompanied by increased levels of members of these transcription factor families. However, the negative regulators of NF‐κB activation, A20/*Tnfaip3,* and inhibitor κB (*Nfkbib*) were found to be highly expressed, possibly resulting in attenuated signal transmission (Fig. 3E). CypD deficiency lowered the number of LPS‐induced DEGs in the TNFR1 (adjPval = 1.85E + 00, ratio = 9/50) and TNFR2 (adjPval = 3.35E + 00, ratio = 9/30) signaling pathways (Fig. 3D,E). The expression of DEGs involved in TNFR pathways among the groups is shown in the heat map in Fig. S2C.

### Regulatory role of CypD‐dependent mPT in NO, ROS, and glutathione‐related pathways

**Fig. 4:**
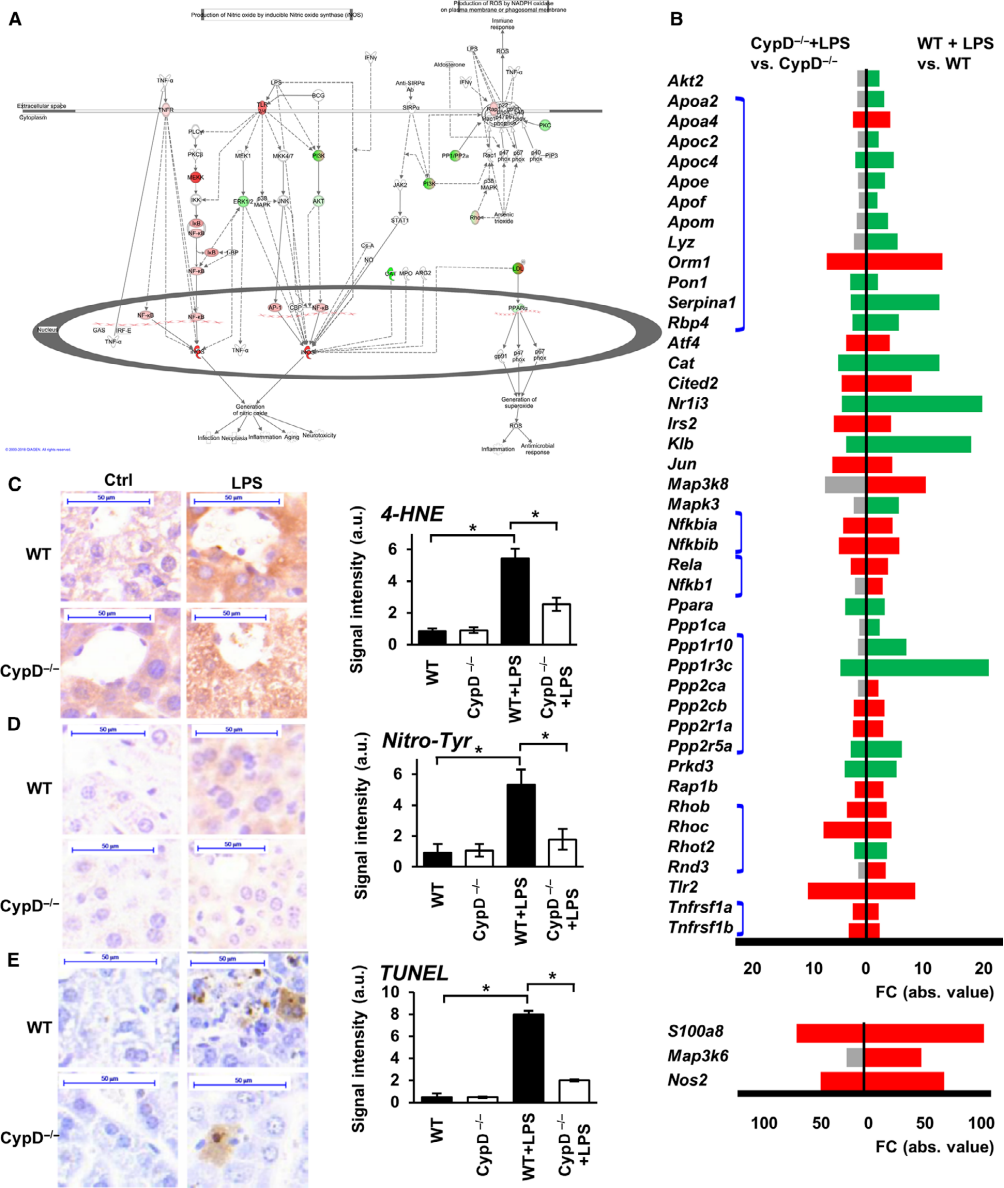
Effect of CypD deficiency on processes of ROS and NO production in LPS‐induced sterile shock. (A) Visualizing DEGs on the production of ROS and NO in macrophages pathway in LPS‐stressed WT animals. Red signs show upregulated genes while green signs represent downregulated genes. Genes with no significant changes labeled with white background. (B) Effect of CypD deficiency on the LPS‐induced DEGs of ROS and NO producing pathway. Red bars show significantly upregulated genes, green bars represent downregulated genes and gray bars show genes not significantly affected. Kal’s *Z*‐test, adjPval < 0.05, [FC] > 1.5. Blue lines connect individual members of complexes (*n* = 5) in order, from top to bottom: LDL compl., I‐κB compl., NF‐κB compl, PP1/PP2a compl., Rho compl., TNF receptor compl.) Please notice, genes with high FC values are presented on a different scale. (C) Effect of CypD deficiency on the LPS‐induced lipid peroxidation characterized by protein‐bound 4‐hydroxynonenal in WT and CypD^−/−^ mouse livers (scalebar = 50 µm). (D) Effect of CypD disruption on the LPS‐induced nitrosylation characterized by the formation of protein nitrotyrosine adducts in WT and CypD^−/−^ mouse livers (scalebar = 50 µm). (E) Effect of CypD deficiency on the LPS‐induced DNA break accumulation determined by TUNEL labeling in WT and CypD^−/−^ mouse livers (scalebar = 50 µm). The bar diagrams with the mean ± SEM of at least three animals are presented, *P < 0.05, one‐way ANOVA followed by Bonferroni *post hoc* comparisons.

Emerging oxidative and nitrosative stress, due to immune cell activity as a consequence of TLR signaling, *Nos2* expression, and mitochondrial dysfunction, play key roles in inflammatory tissue damage following microbial exposure. Twenty‐four hours after LPS exposure, the present analysis demonstrates significant enrichment of DEGs in related pathways in hepatic tissue of WT mice (Fig. 2C, Table S1). Production of NO and ROS in macrophage pathway showed significant enrichment in WT mice following LPS administration (adjPval = 3.81E + 00, ratio = 44/194), leading to a profound increase (more than 60‐fold) in *Nos2* expression (Fig. 4A,B, S1 D), significantly impacting iNOS signaling pathway (adjPval = 1.31E + 00, ratio = 11/45). Conversely, LPS induced suppressed transcription of key antioxidant enzymes, catalase (Cat) and peroxiredoxin 1 (*Prdx1*), and also significantly impacted glutathione‐mediated oxidant defense processes in WT mice, such as glutathione redox reactions I (adjPval = 5.87E + 00, ratio = 14/24), glutathione redox reactions II (adjPval = 1.61E + 00, ratio = 3/4), and glutathione‐mediated detoxification (adjPval = 7.55E + 00, ratio = 18/31) (Fig. 2C, Table S1). These alterations were accompanied by LPS‐induced suppressed expression of the cytoprotective V‐Akt murine thymoma viral oncogene homolog 2 (*Akt2*) and extracellular signal‐regulated kinase 1/2 (*Erk1/2*) genes, which may have exposed hepatocytes to oxidative stress‐induced cell death (Fig. 4A, B and S1D).

**Fig. 5:**
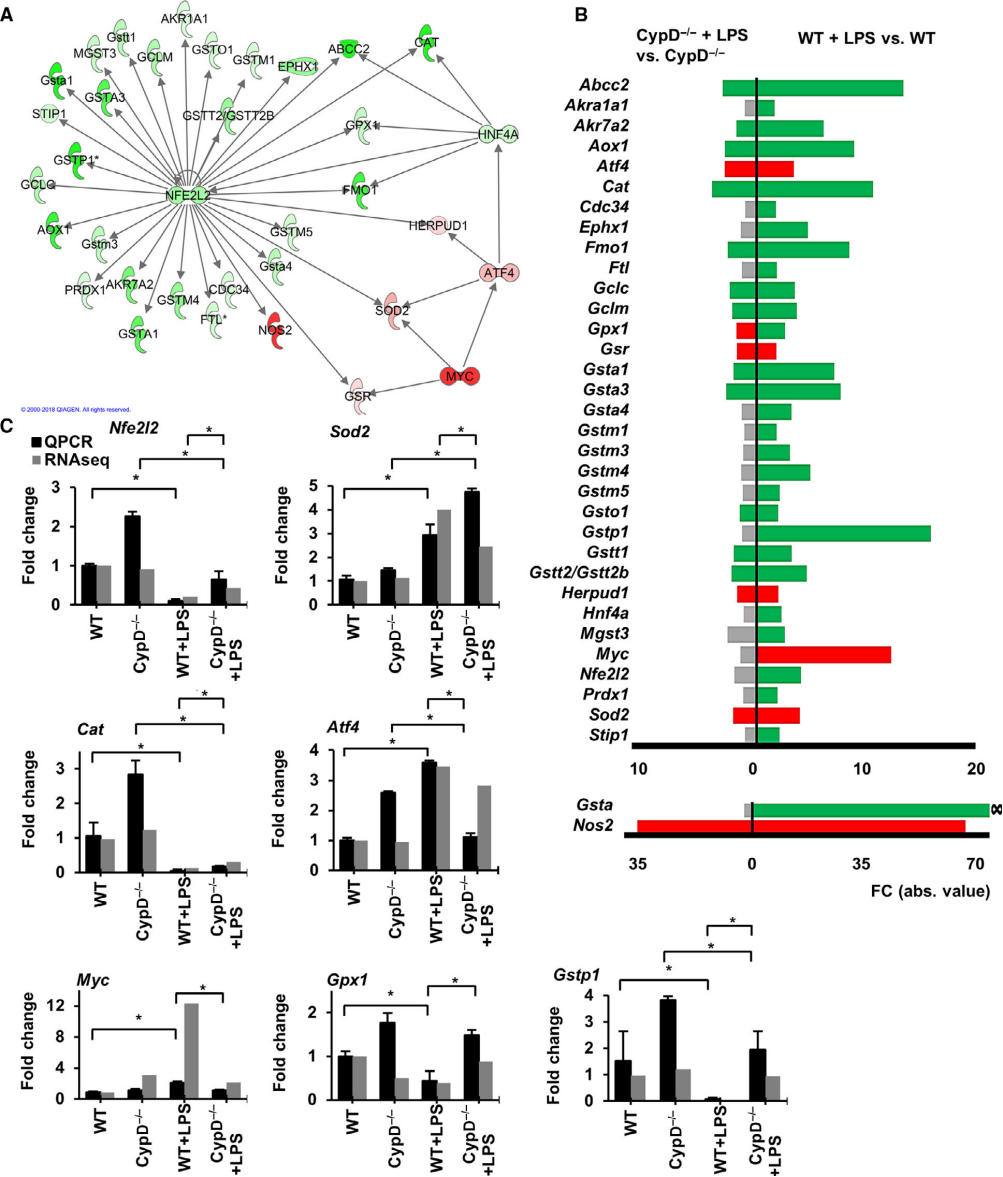
CypD deficiency mitigates the suppressive effect of LPS exposure on Nrf2 regulated antioxidant defense. (A) IPA interaction analysis on the role of Myc and Atf4 in the regulation of Nrf2 target genes at 24 h after LPS exposure in WT mice. Red signs show upregulated genes while green signs represent downregulated genes. (B) Effect of CypD disruption on the LPS‐induced differential expression of Nrf2 target genes involved in glutathione‐mediated antioxidant defense. Red bars show significantly upregulated genes, green bars represent downregulated genes and gray bars show genes not significantly affected. ∞ denotes infinite FC value. Kal’s *Z*‐test, adjPval < 0.05, [FC] > 1.5. Please notice, genes with high FC values are presented on a different scale (*n* = 5). (C) qPCR validation of *Nfe2l2*, *Sod2, Cat, Atf4, Myc, Gpx1,* and *Gstp1* mRNAs levels in sequencing data during LPS‐induced endotoxemia in WT and CypD^−/−^ mice liver. Data are presented as mean ± SEM of FC values normalized to the expression in WT mice, (*n* = 5). *P < 0.05, one‐way ANOVA followed by LSD *post hoc* comparisons on qPCR data.

Disruption of CypD attenuated the effect of LPS on these pathways, leading to a markedly lower production of NO and ROS in macrophages (adjPval = 4.56E + 00, ratio = 30/194) and attenuated iNOS signaling (adjPval = 2.13E + 00, ratio = 9/45) (Fig. 2C). It also preserved glutathione redox reactions I (adjPval = 2.54E + 00, ratio = 7/24) and glutathione‐mediated detoxification (adjPval = 3.97E + 00, ratio = 10/31), while glutathione redox reactions II (adjPval = 4.21E‐01, ratio = 1/4) was not significantly impacted by LPS in CypD‐deficient mice (Fig. 2C, Table S2). Following LPS exposure, a total of 16 genes showed significantly differential expression in the production of NO and ROS in macrophages pathway between WT and CypD^−/−^ mice (Table S3). To further demonstrate the differential expression of subunits of glutathione S‐transferase (*Gstt*), glutathione peroxidase (*Gpx*), and *Akt2* genes among the groups, read alignments are visualized in Fig. S2D (*Gstt1, Gpx4*) or validated by PCR as shown in Fig. 5C (*Gpx1, Gstp1*).

### Effect of CypD deficiency on LPS‐induced lipid peroxidation, protein nitration, and DNA break formation in endotoxic shock

The above data suggest that LPS‐induced alterations in gene expression may lead to elevated levels of oxidative and nitrosative stress (Fig. 4A, B and S1D); therefore, we evaluated the consequences of endotoxic shock in liver tissue by immunohistochemistry. In WT mice, LPS exposure induced accentuated lipid peroxidation, characterized by increased 4‐HNE formation (Fig. 4C). Moreover, a peroxynitrite formation‐related increase in protein tyrosine nitration (Fig. 4D) and oxidation‐induced DNA damage were observed in the liver tissue of WT mice, as visualized by a TUNEL test (Fig. 4E). These data delineate the role of elevated ROS and NO production in LPS‐related tissue damage during the late phase of endotoxemia. Although CypD deficiency in itself had no profound effects on these markers, it reduced the LPS exposure‐related lipid peroxidation (Fig. 4C), nitrotyrosine accumulation (Fig. 4D), and DNA breaks (Fig. 4E), as a possible sign of the reduced amount of ROS and reactive nitrogen species formed following LPS administration in CypD^−/−^ mice. These observations (Fig. 4C–E) reinforce the gene expression data demonstrating the induction of oxidative and nitrosative stress‐related pathways in sepsis, which were attenuated in *CypD* ^−^ *^/^* ^−^ mice (Fig. 4, S1D).

### NRF2‐regulated antioxidant defense

Nrf2 plays an important role in the oxidative stress response during septic shock, since its disruption has been demonstrated to shorten survival and increase mortality in sepsis models, presumably via diminished antioxidant defense mechanisms [25]. However, there are certain contradictions to the expression of *Nrf2* in sepsis models, since its induction has been demonstrated to be dependent on the dose and duration of microbial exposure and on the model applied [26, 27].

Twenty‐four hours after LPS exposure, we observed suppressed expression of *Nrf2* in WT mice, accompanied by a significant enrichment of DEGs in the Nrf2‐mediated oxidative stress response pathway (adjPval = 6.82E + 00, ratio = 52/193) (Fig. 2C, Table S1.). Among its target genes, the LPS‐induced suppressed expression of various forms of glutathione S‐transferases and additional enzymes participating in glutathione biosynthesis and turnover was apparent in WT mice (Fig. 5A,B). Furthermore, the suppressed expression of key antioxidant enzymes, *Cat* and *Prdx1*, may have further propagated oxidative damage of biomolecules in WT mice following LPS exposure assessed by immunohistochemistry (Fig. 4C–E). Additionally, the suppressed expression of *Nrf2* may have facilitated the induction of *Nos2* in WT mice (~60‐fold increase) governed by NF‐κB, further augmenting peroxynitrite formation in the oxidatively stressed liver, as revealed by histology (Fig. 4C–E). Interestingly, in the present study, we also observed elevated expression of Nrf2 target genes, superoxide dismutase 2 (*Sod2)* or glutathione S‐reductase (*Gsr*)*,* which participate in the antioxidant defense, and the expression of these genes may also be governed by the elevated expression and transcriptional activity of activating transcription factor 4 (Atf4) or V‐myc avian myelocytomatosis viral oncogene homolog (c‐Myc) (Fig. 5A–C).

In CypD^−/−^ mice, LPS exposure failed to suppress Nrf2 levels and attenuated the effect on the related pathway (adjPval = 5.50E + 00, ratio = 32/193) and in this manner mitigated the differential induction of the majority of its target genes participating in the glutathione‐mediated oxidative defense (Fig. 5A,B and Fig. S1 E). However, 24 hours after LPS exposure, expression levels of *Sod2* and *Gsr* were found to be elevated, which was accompanied by the increased expression of *Atf4*, similar to that observed in WT mice. The induction of *Nos2* was profoundly attenuated (~30‐fold) in CypD^−/−^ mice, presumably via the inhibitory effect of preserved Nrf2 activity (Fig. 5A–C).

CypD is exclusively found in mitochondria and its inhibition leads to attenuated ROS release from mitochondrial sources in stress scenarios [16, 28]; therefore, we evaluated LPS‐induced differences in the expression of mitochondrial components between WT and CypD^−/−^ mice.

### Nuclear‐encoded mitochondrial genes

**Fig. 6:**
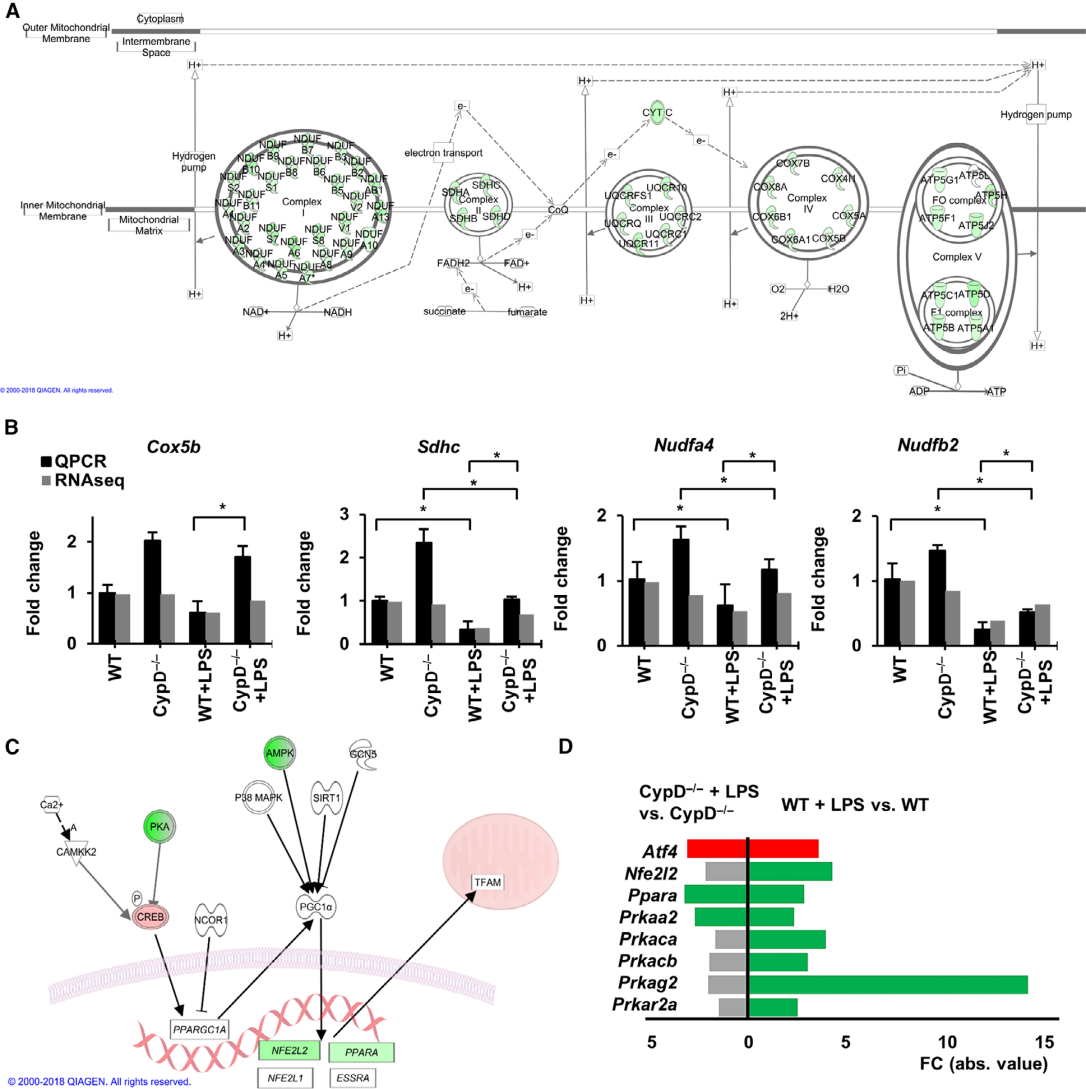
CypD disruption attenuates LPS‐induced alterations of the mitochondrial content. (A) Visualizing LPS induced DEGs on IPA oxidative phosphorylation pathway in WT mice (modified). Green signs represent significantly downregulated gene. Only those genes are shown which were differentially expressed due to LPS exposure. Kal’s *Z*‐test, adjPval < 0.05, [FC] > 1.5. (B) qPCR validation of RNA‐Seq data for *Cox5b, Sdhc, Ndufa4,* and *Ndufb2* mRNA expression. Data are presented as mean ± SEM of FC values normalized to the expression in WT mice (*n* = 5). *P < 0.05, one‐way ANOVA followed by LSD *post hoc* comparisons on qPCR data. (C) Visualizing the LPS‐induced changes on the transcriptional regulators of mitochondrial protein synthesis pathway in WT mice liver. Red signs show significantly upregulated genes while green signs represent significantly downregulated genes and genes with no significant changes labeled white background. (D) Effect of CypD deficiency on the differential expression of regulators of mitochondrial biogenesis pathway. Red bars show significantly upregulated genes, while green bars represent downregulated genes, and gray bars show genes not significantly affected (n = 5) Kal’s *Z*‐test, adjPval < 0.05, [FC] > 1.5.

With respect to nuclear‐encoded mitochondrial components in WT mice, LPS administration induced the suppressed expression of a major portion of the members of mitochondrial electron transfer complexes (Fig. 6A, S3), profoundly impacting the process of oxidative phosphorylation (adjPval = 2.21E + 01, ratio = 57/109), and in this manner propagating mitochondrial dysfunction (adjPval = 2.67E + 01, ratio = 80/171), as revealed by the significant enrichment of DEGs related to these pathways in WT mice (Fig. 2C, S1 F and Table S1). Mitochondrial protection by CypD disruption in our model was delineated by the profoundly lowered numbers of LPS‐affected genes in oxidative phosphorylation (adjPval = 3.12E00, ratio = 18/109) (Fig. 6A, S3) and mitochondrial dysfunction (adjPval = 5.57E00, ratio = 30/171) pathways (Fig. 2C, S1 F and Table S2). Regarding the process of oxidative phosphorylation, CypD deficiency resulted in the differential induction of 24 genes following LPS administration as compared with WT mice (Table S3).

Subsequently, we investigated whether the suppressed expression of various mitochondrial genes may be related to an improper mitochondrial biogenesis in our sepsis model, suggested by the LPS‐induced differential expression of certain complex members regulating this process in WT mice (Fig. 6C, D). Conversely, at the transcriptional level, we found no significant enrichment of LPS‐affected DEGs in the Kyoto Encyclopedia of Genes and Genome’s mitochondrial biogenesis pathway gene set in any of the comparisons: (adjPval = 2.95E‐01, ratio = 8/53) or (adjPval = 4.21E‐01, ratio = 4/53) for LPS‐stressed WT or CypD^−/−^ mice, respectively. To further demonstrate the differential expression of the genes encoding components of respiratory complexes, NADH:ubiquinone oxidoreductase subunit B5 (*Ndufb5*), cytochrome C oxidase subunit 7C (*Cox7c*), and succinate dehydrogenase complex subunit C (*Sdhc*), read alignments are visualized in Fig. S2 E. RNA‐seq data were validated by qPCR, as presented in Fig. 6B for various subunits.

### Mitochondrial‐encoded mitochondrial genes

**Fig. 7:**
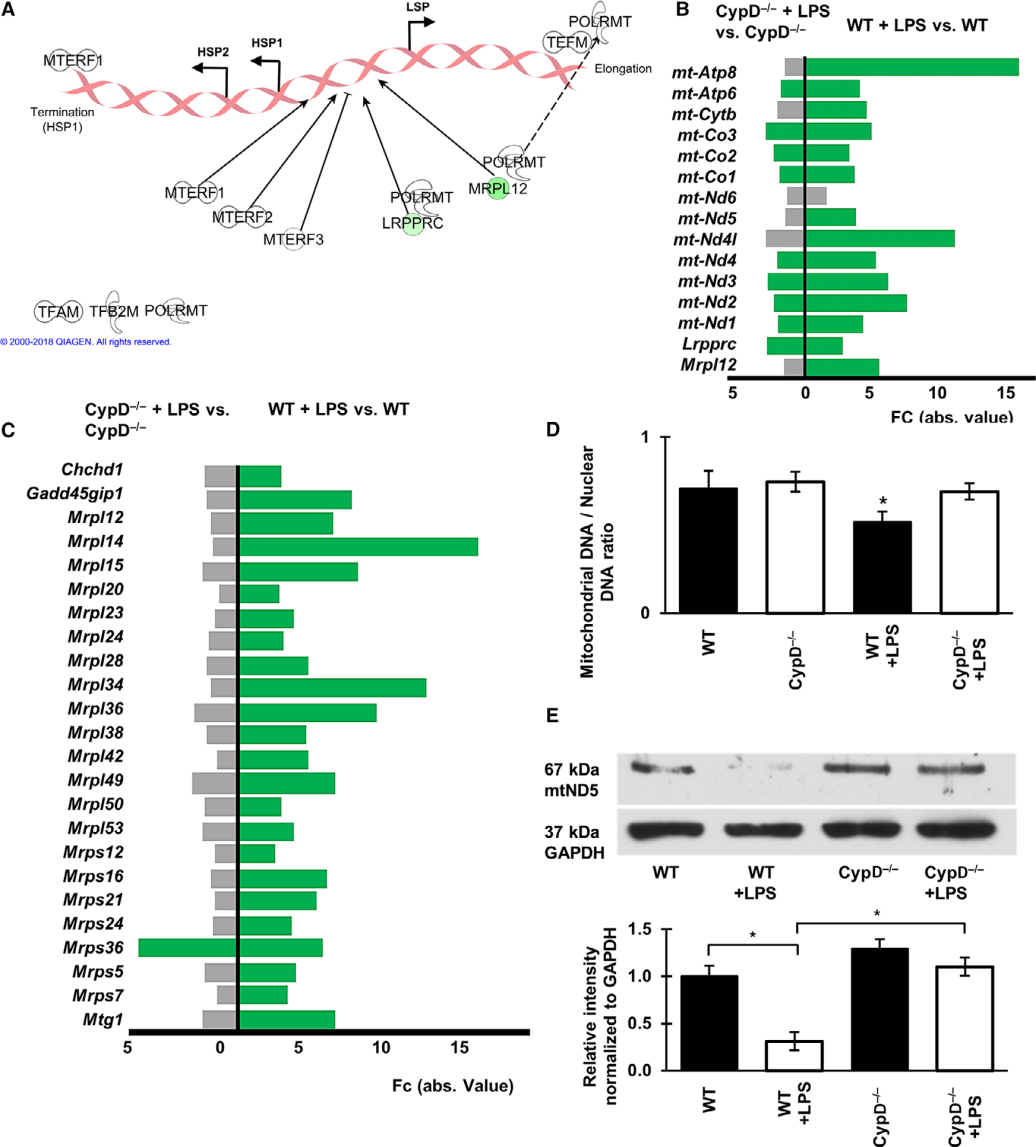
CypD deficiency attenuates LPS‐induced alterations regarding processes of mitochondrial replication in the sterile shock model. (A) Effect of CypD deficiency on the LPS‐induced alterations of regulators of transcription from mtDNA. Green symbols represent downregulated genes, while white symbols show genes not significantly affected. (B) Fold change values of LPS‐induced DEGs in the mitochondrial transcription regulators and mtDNA‐encoded genes in WT and CypD^−/−^ mice. Green bars represent downregulated genes gray bars show genes not significantly affected, (*n* = 5), Kal’s *Z*‐test, adjPval < 0.05, [FC] > 1.5. (C) Bar graph demonstrating DEGs for mitochondrial ribosomes and accessory proteins in LPS‐exposed WT and CypD^−/−^ mouse liver tissues. Green bars represent downregulated genes gray bars show genes not significantly affected, (*n* = 5), Kal’s *Z*‐test, adjPval < 0.05, [FC] > 1.5. (D) The effect of CypD disruption on LPS‐induced changes of mtDNA levels relative to nuclear DNA content in liver tissues during endotoxemia, determined by qPCR. Data are presented as mean ± SEM, (*n* = 5), *P < 0.05, one‐way ANOVA followed by Bonferroni *post hoc* comparisons. (E) Western blot analysis of the effect of CypD disruption after LPS exposure on the cellular level of mitochondrial protein complex I component, mtND5. Data are presented as mean ± SEM (*n* = 5), *P < 0.05, one‐way ANOVA followed by Bonferroni *post hoc* comparisons.

Transcription of mitochondrial‐encoded mitochondrial genes is regulated by a different set of transcription factors and regulators than that of nuclear‐encoded mitochondrial genes [29] (Fig. 7A). In WT mice, LPS suppressed the expression of leucine‐rich pentatricopeptide repeat‐containing (*Lrpprc*) and mitochondrial ribosomal protein L12 (*Mrpl12*), which are involved in the regulation of transcription and translation initiation of mitochondrial‐encoded mitochondrial genes (Fig. 7A,B). Moreover, in our model, the suppressed expression following LPS administration was apparent for components of respiratory complexes encoded in mtDNA (Fig. 7B), similar to those transcribed from the nuclear genome (Figs 6A, S3). Additionally, assessment of the mtDNA content by qPCR analysis revealed a marked decrease in the mtDNA: nuclear DNA ratio in LPS‐exposed WT mice, while this effect was nonsignificant in CypD^−/−^ mice (Fig. 7D).

To evaluate whether the suppressed expression of mitochondrial components, encoded by both genomic DNAs, and the LPS‐related decrease in mtDNA content may also impact the translational machinery, the expression patterns of mitochondrial ribosomal proteins (Mrpl) and accessory proteins among the groups were assessed (Fig. 7C). In WT mice, LPS administration induced a marked decrease in expression of a major portion of these genes, but in CypD^−/−^ mice, ribosomal protein content was more preserved. While the lack of CypD itself induced a mild suppression of the expression of *Mrpl*‐12 and −14 by −1.597‐ and −2.298‐fold, respectively, LPS exposure led to differential expression of only one gene in CypD^−/−^ mice. Conversely, LPS administration in WT mice induced significantly lower expression of 24 ribosomal proteins in this gene set (Fig. 7C).

In accordance with the observations regarding the transcription and translation processes of mitochondrial components at the protein level, western blot analysis of mitochondrial respiratory chain complex I 67 kDa component, mtND5 indicated reduced cellular levels in LPS‐exposed WT mice, which was not observed in CypD^−/−^ mice (Fig. 7E). These findings demonstrate that disruption of CypD, and thus the prevention of mPT, attenuates the LPS‐induced alterations in nuclear‐encoded mitochondrial gene expression and also the ability to protect processes of mitochondrial replication, transcription, and translation in our endotoxic shock model.

## Discussion

CypD‐dependent mPT plays a significant role in the progression of ischemic heart [12, 13] and neurodegenerative diseases [30]; however, its role in sepsis or the regulation of inflammatory gene expression is not well understood. Cyclosporin A is the best‐studied mPT inhibitor, which blocks the action of cyclophilins and calcineurin and regulates several extramitochondrial pathways [16, 31]. It is well‐known that inhibitors of calcineurin/NFAT binding, such as cyclosporin A and FK506 are widely used in organ transplantation and can act as potent immunosuppressive drugs in a variety of diseases [31]. In endotoxemia, both these molecules have a protective effect in spite of the fact that FK506 is not a direct mPT inhibitor, showing a possible nonmitochondrial protective mechanism, namely the inhibition of calcineurin [31]. Therefore, cyclosporin A has not provided direct evidence for the role of mPT in septic shock models.

Inflammatory pathways and reactive oxygen/nitrogen species synthesis or metabolism are critically important in liver tissue during sepsis, since oxidative liver damage can further initiate production of TNFα, interleukins, and chemokines by immune cells [18]. The induced expression of these mediators can augment inflammation in the liver characterized by ROS production, creating a vicious cycle that may lead to organ failure and fatality. The attenuated oxidative/nitrosative damage of liver tissue in CypD‐deficient mice, 24 h after LPS exposure (Fig. 4C–E), was in accordance to our previous observations in lung endotoxemia, where the more preserved tissue structure was accompanied by lower neutrophil infiltration [16]. Also, disruption of CypD suppressed the activation of NF‐κB and the morphological characteristics of macrophage activation after LPS administration, in vitro [17]. Governed by cytokine production, these observations could be explained by the attenuated TLR (Fig. 3A, B) and TNFR signaling (Fig. 3D, E), and lowered NO and ROS production in macrophages (Fig. 4A, B) revealed by IPA analysis, in the liver tissue of CypD‐deficient endotoxemic mice.

During inflammatory response, p38 mitogen‐activated protein kinase (MAPK) regulates NF‐κB and immune cell activation and additionally propagates cell death by destabilizing the mitochondrial membranes. We previously demonstrated that in case of oxidative stress and the consequent DNA damage response, the ongoing nuclear events, especially the activation of poly(ADP‐ribose)‐polymerase 1 is able to attenuate the negative feedback on MAPK activity via suppressed MAPK phosphatase‐1 expression [32, 33]. Here we propose that via a mitigated mitochondrial ROS accumulation, disruption of CypD is able to attenuate the emerging collapse of energy metabolism and mitochondrial dysfunction. [32] By lowering mitochondrial alterations and tissue damage due to LPS administration, CypD deficiency prevents a vicious cycle leading to systemic failure. Here, we tested this hypothesis and analyzed the role of CypD‐dependent mPT on liver gene expression in LPS‐induced endotoxic shock model.

The present RNA‐seq data show surprisingly large and wide‐range effects of CypD‐dependent mPT on LPS‐induced inflammatory reprogramming (Fig. 2A–C). CypD disruption reduced the number of LPS‐induced genes by ~ 50% and altered the activatory or suppressive effects of LPS on a large number of signaling and metabolic pathways (Fig. 2C, S1A, Tables [Link], [Link], [Link], S2, S3). Several studies have explored LPS‐induced reprogramming in macrophages [6, 22, 23, 34]; however, much less data are available for inflammatory reprogramming in hepatocytes or liver tissue in a sepsis model [21, 35]. Here, we provide evidence for the large‐scale reprogramming of gene expression in LPS‐induced endotoxic shock, affecting 2715 genes and 179 pathways, as determined by IPA^®^ (Table S1). The differential expression of 1369 genes indicates that the disruption of CypD significantly reduced LPS‐induced alterations (Fig. 2C). Additionally, the great majority of DEGs related to CypD disruption was also affected by LPS exposure, which left only 16 DEGs unique to the CypD^−/−^ vs WT comparison (Fig. 2B). Pathway analysis by IPA^®^ showed that LPS induced significant changes in pathways affecting inflammatory and oxidative stress‐related processes (Fig. 2C, Table S3), which were attenuated by the disruption of CypD, demonstrating its protective role regarding the inflammatory reprogramming during endotoxic shock.

In addition to inflammatory pathways, oxidative stress plays an important role in multiple organ failure and survival [32, 33]; therefore, we analyzed the consequences of CypD deficiency in the reprogramming of oxidative stress‐related pathways. LPS treatment reduced the expression of *Nrf2* and Nrf2‐regulated genes, leading to a compromised function of key antioxidant enzymes, such as *Cat* and *Prdx1*, and also significantly impacted glutathione‐mediated oxidant defense system in the liver of WT mice (Fig. 5A,B, S1E). These changes in the antioxidant system contribute to and indicate mitochondrial dysfunction, propagating further production of ROS and oxidative stress leading to protein, DNA and tissue damage (Fig. 4C–E). Emerging oxidative stress accompanied by accentuated peroxynitrite formation in face of a somewhat diminished cellular antioxidant defense may lead to excess damage of the mitochondrial population, propagating its quality control mechanisms. Moreover, proper Nrf2 function and physiological NO and ROS production is required for the processes of mitochondrial biogenesis. Disruption of CypD attenuated the effect of LPS on these pathways, leading to a markedly lower production of NO and ROS and less severe oxidative stress‐related tissue damage (Fig. 4C–E) which is one of the main causes of multiple organ failure and fatality.

Damage and dysfunction of mitochondria are observed in septic shock, and it is considered an important factor in the induction of multi‐organ failure during endotoxemia [7, 8, 36]; however, in sepsis models, changes in mitochondrial biogenesis are contradictory, since compensatory increases in the rate of mitochondrial biogenesis occurs in some cases [37, 38], while the suppression of the rate of mitochondrial biogenesis is observed in other cases [39, 40]. It is a significant observation that stabilization of the mitochondrial membrane system, by preventing CypD‐dependent mPT, attenuated LPS‐induced mitochondrial dysfunction and the suppressed expression of members of the oxidative phosphorylation complexes (Figs 6A, S3, Table S1, S2). Although NO and ROS, generated by the inflammatory response induce mitochondrial damage, they can also contribute to the activation of mitochondrial biogenesis [41]; therefore, it is likely that shifting NO and ROS production toward the physiological range, CypD disruption can also protect mitochondrial biogenesis in other ways, perhaps via the mitigation of LPS‐induced suppression of *Nrf2* levels and cellular antioxidant defense system (Fig. 5A, B). Data regarding LPS‐suppressed mitochondrial biogenesis have been published elsewhere [39], and data from pediatric sepsis patients have shown a reduction in the expression of mitochondrial proteins encoded by nuclear genes in sepsis [40]. Sepsis‐induced suppression of mitochondrial biogenesis is considered an important factor in fatality [40]; therefore, our observation that inhibition of mPT attenuates the inflammatory suppression of mitochondrial biogenesis indicates that CypD (CypD‐dependent mPT) may be a therapeutic target in endotoxic shock.

Here, we used an LPS‐induced endotoxemic model, and the serious question was how well this model could represent the complex gene expression changes in human sepsis or septic shock conditions. Recently, there have been attempts to differentiate between sepsis patients who survive and those who die, and the main difference between these groups was the occurrence of mitochondrial dysfunction and impairment of oxidative phosphorylation pathways [40]. In our model, WT mice died in the immunosuppressive phase (Fig. 1A, C), and similar to nonsurviving humans in sepsis, they were characterized by mitochondrial dysfunction and impaired oxidative phosphorylation (Figs 2C, 6A, S3). Surviving sepsis patients have more preserved mitochondrial function [42], similar to the CypD^−/−^ mice, showing that the prevention of mitochondrial mPT could contribute to the better survival. Furthermore, the present data show a correlation between the expression levels of nuclear‐ and mitochondrial‐encoded components of respiratory complexes (Figs 6A, 7B, S1F, S3), despite the fact that their transcriptional regulation is different. CypD disruption attenuated the LPS‐induced suppression of nuclear‐encoded mitochondrial translation machinery components (Fig. 7C) and mtDNA‐encoded gene expression (Fig. 7B), showing a protective effect on both genomes. These are the first data showing that the prevention of CypD‐dependent mPT attenuates the LPS‐induced suppression of nuclear‐ and mitochondrial‐encoded mitochondrial genes, including mitochondrial transcription machinery genes in endotoxic shock.

Disruption of CypD prevents mPT and therefore attenuates LPS‐induced complex changes, resulting in the maintenance of more stable mitochondrial structure and less ROS‐related signaling. The most dramatic effects of CypD disruption were the reduction of LPS‐induced reprogramming and the protection of mitochondria and complexes of oxidative phosphorylation (Fig. 6A, S1F, S3), which must have an important effect on the survival of the mice (Fig. 1C). Therefore, CypD‐dependent mPT inhibition, which mitigates mitochondrial dysfunction, can be a novel drug target in endotoxic shock and other inflammatory diseases.

## Materials and methods

### Animals

Male C57BL/6 mice, purchased from Charles River Budapest, Hungary Breeding and male *Ppif* ^‐/−^ (CypD^−/−^) mice of C57BL/6 background, supplied by Prof. László Tretter (Semmelweis University, Budapest, Hungary) were used for the experiments. All animals were 16–20 weeks old, weighting 20–30 g. Tap water and mouse chow were provided ad libitum. Mice were kept under standard conditions according to the Guidelines for the Care and Use of Laboratory Animals published by the US NIH, and the experiment was approved by the Animal Research Review Committee of the University of Pecs, Medical School.

### Materials

Lipopolysaccharide from Escherichia coli 0127:B8 (Sigma‐Aldrich Budapest, Hungary; L 8654). The following primary antibodies were applied: NADH dehydrogenase subunit 5 (mtND5) (Thermo Fisher Scientific, Waltham, MA USA; PA5‐36600), glyceraldehyde‐3‐phosphate dehydrogenase (Gapdh) (Merck KGaA, Darmstadt, Germany; #AB2302), and nitrotyrosine clone 1A6 (Merck KGaA, Darmstadt, Germany; #05‐233) and 4‐Hydroxynonenal (4‐HNE) (Merck KGaA; #AB5605). To label the fragmentation of nuclear DNA, ApopTag^®^ Plus Peroxidase In Situ Apoptosis Detection Kit (Merck KGaA; #S7101) was used according to the manufacturer's instructions.

### Experimental setup

C57BL/6 wild‐type and CypD^−/−^ mice were randomized into two groups. To induce murine endotoxic shock, a single dose of intraperitoneal LPS (40 mg·kg^−1^, dissolved in PBS) was given to the LPS group while the control groups received PBS (10 µL·g^−1^) intraperitoneally. Twenty‐four hours after treatment, all mice were sacrificed by overexposure to isoflurane (Isopharma, Pasig City, Philippines). Abdomen was opened, and liver was removed. Different samples were (i) fixed in 10% paraformaldehyde (pH 7.2), (ii) snap frozen in liquid N_2_, (iii) submerged into RNALater (QIAGEN GmbH, Hilden, Germany; ID: 76104) RNA stabilization reagent, or (iv) homogenized as described further on.

### mRNA isolation from liver tissue and quantitative reverse polymerase chain reaction (qPCR)

Total mRNA was isolated from tissue samples [20] using RNeasy Mini Kit (QIAGEN GmbH; ID: 76106) according to the manufacturer’s instructions. To reach higher RNA yields QIAshredder (QIAGEN GmbH; ID: 79656), homogenizer was used. The quality of extracted RNA was determined using Bioanalyzer 2100 (Agilent Technologies Santa Clara, California, USA) measurements. Total RNA concentration was determined using spectrophotometric method (IMPLEN NanoPhotometer^TM^) or Qubit RNA Broad‐range assay kit (Thermo Fisher Scientific, Waltham, MA USA; Q12210) and reverse‐transcribed into cDNA with either RevertAid First Strand cDNA Synthesis Kit (Thermo Fisher Scientific, Waltham, MA USA; K1621) or SuperScript VILO Master Mix (Thermo Fisher Scientific, Waltham, MA USA; 11755050) according to the manufacturer’s instructions. SybrGreen technology‐based real‐time quantitative PCR was used to quantify the relative abundance of the selected mRNAs. For this, specific exon spanning gene expression assays were used (Table 1). As controls, we used reaction mixtures without cDNA. All of the measurements were performed in duplicate with at least three biological replicates. The ratio of each mRNA relative to the beta‐actin (Thermo Fisher Scientific, Waltham, MA USA; assay ID: Hs99999901) was calculated using the 2^‐ΔΔ^ *^C^* ^T^ method.

**Table 1:** Sequences of PCR primers used in the study

Gene	Forward (5'‐3')	Reverse (5'‐3')
*Atf4*	TTA ATA AAA GTC GAC CAG GTT GCC	GAG TGT CTT CCT CCT TTA CAC ATG G
B2Microglobulin	AAA TTC CTA AAG TAG AGA TGT	AGG CGT ATG TAT CAG TCT CAG TGG
*Cat*	TTTTGCCTACCCGGACACTC	GGGGTAATAGTTGGGGGCAC
*Cd14*	CCTGAATTGGGCGAGAGAGG	GCATCCCGCAGTGAATTGTG
c‐Jun	CCT GTC CCC TAT CGA CAT GG	GGA GTT TTG CGC TTT CAA GG
*c‐Myc*	TAG TGA TCC TCA AAA AAG CCA CC	TCA GTT TAT GCA CCA GAG TTT CG
*Cox5b*	GGGCTGGAGAGGGAGATCAT	TGCTGATGGACGGGACTAGA
*Csf1*	ACA TAA TAG ATG AGA CCA TGC GC	TTC TTG ATC TTC TCC AGC AGC
*Cxcl2*	*GCT GTC AAT GCC TGA AGA CC*	*GGA TGA TTT TCT GAA CCA GGG*
*Foxo1*	CTT GGA CTG TGA CAT GGA GTC C	AGT CTT GAC ACT GTG TGG GAA GC
*Gpx1*	TGCAATCAGTTCGGACACCA	GGAAGGTAAAGAGCGGGTGA
*Gstp1*	GCGGCAAATATGTCACCCTC	CGAGCCACATAGGCAGAGAG
*Hif1α*	CAA TGT CTC CTT TAC CTT CAT CGG	CAC ATC AAA GCA ATA TTC ACT GGG
*Ifng*	GCG GCG AGA ACG AGA AGA A	GGG GAA GTG GCA ACT GAT GA
Mito12SrRNA	CTT AAA ACT CAA AGG ACT TGG CG	TGG CGG TAT ATA GGC TGA ATT AGC
*Ndufa4*	AGCATCCCAGCTTGATTCCTC	TGCCAAGCGCATCACATACA
*Ndufb2*	GGGAGTTTCCCCAGCTTACC	ACCTTGCTCCTACTCGTCCA
*Nfe2l2*	GGACATGGAGCAAGTTTGGC	CCAGCGAGGAGATCGATGAG
*Nf‐kB1*	TAT CGT TCA GTT GGT CAC AAA TGG	TGT AGA TAG GCA AGG TCA GAA TGC
*Nf‐kB2*	ATC CAT GCA GAG AAT GAG GAG C	CGG AAG TTT CTT TGG GTA TCC C
*Rela*	ATTGCTGTGCCTACCCGAAA	TACCATGGCTGAGGAAGGGA
*Rhot2*	AGGTGCCCACTCATATCGTG	TGCCATTCACAAGCGGGAT
*Sdhc*	AGCTTTGTATCAGAAATGCTGCTC	GCTATTCCAGAGCCTCGGTG
*Sod2*	AATCAGGACCCATTGCAAGGA	TGAAGGTAGTAAGCGTGCTCC
*Stat3*	AAA GGA CAT CAG TGG CAA GAC C	AGT GGA GAC ACC AGG ATG TTG G
*Tank*	GCA ACT CAA TAG AGC ATA TGA AGC C	TCA TCA AGG GTC AAA TTA TTC TTC C
*Tbk1*	ATC TGT GGC TCC TGT CTG ATA TCC	GCA CTT TAT GTC TTG TTG TTG TCT CC
*Tlr2*	GAA GCG AAT CAC AGT AGA GAA CAG C	CTA AGG TTT GTA GAG AAG GCC AGG
*Tnfa*	ATG AGC ACA GAA AGC ATG ATC	TCA CAG AGC AAT GAC TCC AA

### Whole transcriptome sequencing

Whole transcriptome sequencing was performed as described previously [43]. Briefly, RNA quality and quantity measurements were performed on Bioanalyzer 2100 (Agilent Technologies Santa Clara, California, USA) and Qubit (Thermo Fisher Scientific, Waltham, MA USA; Q12210). High‐quality (RIN > 8.5) total RNA samples from three biological replicates were pooled in equimolar concentrations and processed using the SOLiD total RNA‐Seq Kit (Thermo Fisher Scientific, Waltham, MA USA; 4445374) according to the manufacturer’s instructions. For this, 5µg of RNA was DNaseI (18068015) treated and the ribosomal RNA depleted using RiboMinus Eucaryote kit for RNA‐Seq (A1083708) and RiboMinus Concentration Module (K155005) all three from Thermo Fisher Scientific, Waltham, MA USA. The leftover was fragmented using RNAseIII (part of the kit), the 50‐200 nt fraction size‐selected, sequencing adaptors ligated and the templates reverse‐transcribed using ArrayScript reverse transcriptase (part of the kit). The cDNA library was purified with Qiagen MinElute PCR Purification Kit (QIAGEN GmbH; ID: 28006) and size‐selected on a 6% TBE‐Urea denaturing polyacrylamide gel (Thermo Fisher Scientific, Waltham, MA USA; EC6865BOX). The 150‐250 nt cDNA fraction was amplified using AmpliTaq polymerase (Thermo Fisher Scientific, Waltham, MA USA; N8080171) and purified by AmPureXP Beads (Agencourt, Beckman Coulter GmbH Krefeld, Deutschland; A63882). Concentration of each library was determined using the SOLiID Library TaqMan Quantitation Kit (Thermo Fisher Scientific, Waltham, MA USA; 4449639, A12127, and A12126). Each library was clonally amplified on SOLiD P1 DNA Beads by emulsion PCR (ePCR) (Thermo Fisher Scientific, Waltham, MA USA; 4400834). Emulsions were broken with butanol (Thermo Fisher Scientific, Waltham, MA USA; 4389770) and ePCR beads enriched for template‐positive beads by hybridization with magnetic enrichment beads (Thermo Fisher Scientific, Waltham, MA USA; 4387894). Template‐enriched beads were extended at the 3' end in the presence of terminal transferase and 3' bead linker. Beads with the clonally amplified DNA were deposited onto SOLiD flowchip (Thermo Fisher Scientific, Waltham, MA USA; 4461826) and sequenced on SOLiD 5500xl System using the 75‐base sequencing chemistry (Thermo Fisher Scientific, Waltham, MA USA; 4473598). Bioinformatic analysis of the whole transcriptome sequencing was performed in color space using Genomics Workbench v6.0.1 (CLC Bio, now part of Qiagen). Raw sequencing data were trimmed by removal of low quality, short sequences so that only 75 nucleotide long sequences were used in further analysis. Sequences were mapped in a strand specific way onto the *Mus musculus* GRCm38 genome, using default parameters except for the following: minimum length 50%, minimum similarity 80% with the unspecific match limit set to 10. To identify significantly altered gene expressions in between samples, we used Kal’s Z‐test with false discovery rate (FDR) correction. Clustering was done by Hierarchical Clustering module available in the GenePattern suite [44, 45]. The gene enrichment and pathway analyses were done through the use of QIAGEN’s Ingenuity Pathway Analysis (IPA^®^, QIAGEN Redwood City, http://www.qiagen.com/ingenuity).

### mRNA profile

The gene expression changes were considered as significant if their fold changed value was greater than 1.5x in either direction and the FDR corrected statistical significance was lower than 0.05. These set of differentially expressed genes (DEGs) were post processed through the use of IPA^®^ to find the major biological processes associated to the whole transcriptome analysis performed. The overrepresentation of DEGs was tested in the Canonical Pathway section of IPA^®^. Selected pathway diagrams were plotted in a way that the data set molecules which met the filtering cutoff criteria of significant up or down regulation were colored in red or green, respectively. The color intensity applied was proportional with the level of gene expression alteration below the fold changed value of 10x. Description of signs and labels used in the diagrams can be downloaded from Ingenuity homepage. For pathway analyses, the ‐log of adjusted p‐values by Benjamini–Hochberg methodology (adjPval) and ratios are demonstrated, the latter in the form of: (#of DEGs)/(# of genes designated to the given canonical pathway in IPA^®^). Hierarchical clustering and heat map representation of the significant members of the pathways were done in GenePattern.

### The mtDNA and nuclear DNA quantitation by QPCR

The method is based on the Ajaz’s publication [46]. Briefly, DNA from mice liver was extracted by Nucleo‐Spin Tissue kit (MACHEREY‐NAGEL GmbH & Co. KG, Düren, Germany; ID: 740952.250) and the concentrations were measured. The concentrations were adjusted to 5 ng·µL^−1^. 1st part: Calibration curve. The samples were amplified by β2 microglobulin (β2m) and mitochondrial 12S rRNA (mito12SrRNA) primers. The products were checked by electrophoresis. Excised bands were purified by QIAquick Gel Extraction Kit (QIAGEN GmbH, Hilden, Germany; ID: 28706) according to the manufacturer’s instructions, eluted in 25 µL of elution buffer, and the concentrations were determined. Copy numbers were calculated by the following formula: copy number·µL^−1^ = [(ng·µL^−1^)*(1/mol)]/[(length bp*660 g·mol^−1^ *109 ng·g^−1^)]. β2m and mito12SrRNA PCR products were diluted containing 109 copies per µl of the DNA standard stock solution (SSS). Each SSS was diluted in tenfold for 2–8 log. PCR was performed from each sample to determine the correlation between the Ct and copy number (calibration curve). 2nd part: Absolute quantification. The template DNA was used for qPCR to determine the amount of mtDNA and nuclear DNA in the sample. Determination of Ct value gives back the concentration (or copy number) by the calibration curve. Template DNA and SSS DNA have run parallel way. 3rd part: Mt/Nuclear DNA ratio is given by dividing the average of copy number of Mt samples, and the average of the copy number of the nuclear DNA.

### Immunohistochemistry

Immunohistochemistry was performed on tissue sections. The sections were probed with antibodies indicated in the figures and listed in the materials and methods section for 1 h at room temperature. Secondary antibodies (HISTOLS® ‐R Detection System, Rabbit, # 30011.R500, HISTOLS® ‐M Detection System, Mouse, # 30011.M500; Histopathology Ltd., Budapest, Hungary) were applied for 30 min at room temperature. Sections were incubated with 3‐amino‐9‐ethylcarbazol (HISTOLS^®^ ‐Resistant AEC, # 30015.K, Histopathology Ltd., Budapest, Hungary) and counterstained with hematoxylin.

### Western blot analysis

Ten mg of frozen tissue was homogenized with ultra turrax and Potter homogenizer in 150 µL lysis buffer (50 mm TRIS, 50 mm EDTA, 50 mm sodium metavanadate, 0.5% protease inhibitor cocktail (Sigma‐Aldrich, Budapest, Hungary; I3786) and 0.5% phosphatase inhibitor cocktail (Sigma‐Aldrich; 524627), pH 7.4). The cell homogenate was sonicated, and the protein concentration was determined with protein assay kit (Bio‐Rad, Budapest, Hungary; #500‐0114) based on Bradford’s method according to the manufacturer’s description. The cell lysate was diluted in Laemmli buffer, boiled for five minutes and centrifuged (10 000 ***g***, 7 min), and the clear supernatant was used for further investigations. Tissue extracts were separated with SDS/PAGE with protein loads of 20 µg/lane and transferred onto nitrocellulose membrane. The membranes were blocked with 5% nonfat dried‐milk proteins in TBS and 0.1% Tween, incubated with the primary antibody at 4 °C overnight at a dilution of 1:1000. The secondary antibody was horseradish peroxidase‐conjugated goat anti‐rabbit IgG (Sigma‐Aldrich; A6154). Peroxidase labeling was visualized with the ECL Western blotting detection system (Amersham Bioscience, GE Healthcare Bio‐Sciences, Pittsburgh, PA, USA; RPN2232). Quantification of band intensities of the blots was performed in imagej software (National Institute of Mental Health, Bethesda, MD, USA).

### Statistical analysis

Statistical evaluations were performed using the ibm spss (Armonk, NY, USA) Statistics program for Windows, v21.0. Graphs were plotted with GraphPad Prism 6 software (San Diego, CA, USA) or in Microsoft Excel 2016. Quantitative data are presented as the mean ± SEM. The significance of difference between sets of data was determined by independent samples t‐test or by one‐way analysis of variance (ANOVA) following Bonferroni or LSD post hoc test; a *P* value of less than 0.05 was considered significant.

## Conflict of Interest

The authors declare no conflict of interest.

## Authors' contributions

BV, FG, and BS supervised the project, designed and performed experiments, and wrote the paper. CA and NK performed Q‐RT‐PCR. FF and PBJ performed the in vivo studies the histological and western blot experiments. KE, EB, ZH, and IN performed the gene expression analysis, statistical evaluations, and data interpretation. LT provided and screened mouse lines.

## Supporting information


**Fig S1.** Heat maps demonstrating expression levels of individual genes among the study groups, (n=5).

**Fig S2.** Read coverage patterns derived from RNA‐seq experiments in mice liver tissue.

**Fig S3.** Effect of CypD disruption on the LPS induced differential expression of nuclear DNA encoded genes of oxidative phosphorylation.

**Table S1.** LPS induced significant enrichment of DEGs in WT mice liver tissue in canonical pathways analyzed by IPA. ‐lg(adjPval.) > 1.3.

**Table S2.** LPS induced significant enrichment of DEGs in CypD^‐/‐^ mice liver tissue in canonical pathways analyzed by IPA. ‐lg(adjPval.) > 1.3.

**Table S3.** List of DEGs in the CypD^‐/‐^+LPS vs WT+LPS comparison in canonical pathways analyzed by IPA. ‐lg(adjPval.) > 1.3.

## Data Availability

Gene Expression Omnibus (GEO) archive of the four sequenced libraries was deposited in NCBI’s GEO Archive at http://www.ncbi.nlm.nih.gov/geo under accession GSE79059.
