# Supplementary figures and images for: Cyclophilin D‐dependent mitochondrial permeability transition amplifies inflammatory reprogramming in endotoxemia
Source: FEBS Open Bio. 2021 Feb 13;11(3):684–704. doi: 10.1002/2211-5463.13091 (PMC7931201; doi:10.1002/2211-5463.13091)

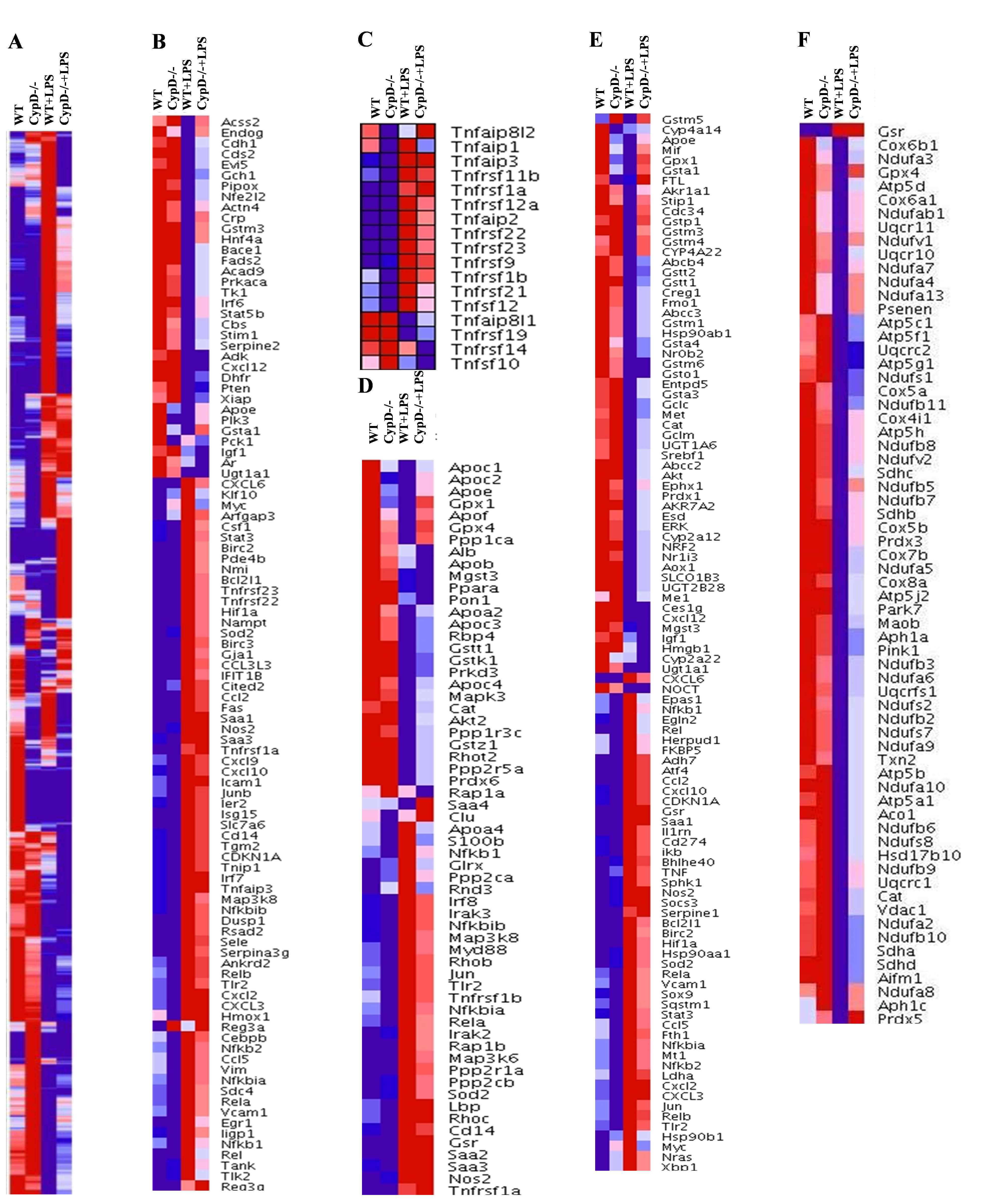

Supplement: Supplementary file 1 — Fig S1. Heat maps demonstrating expression levels of individual genes among the study groups, (n=5). [file FEB4-11-684-s001.tif]

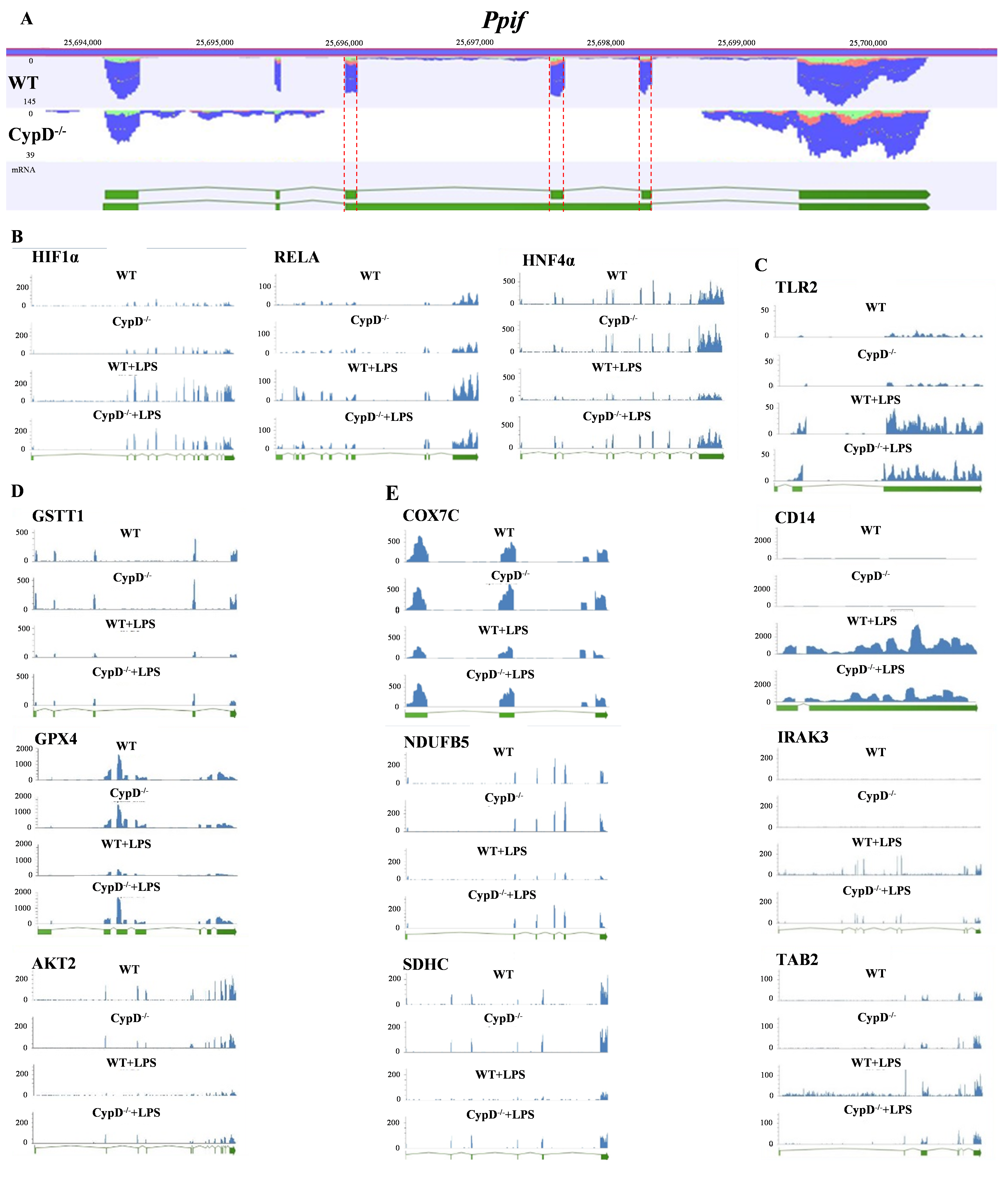

Supplement: Supplementary file 2 — Fig S2. Read coverage patterns derived from RNA‐seq experiments in mice liver tissue. [file FEB4-11-684-s002.tif]

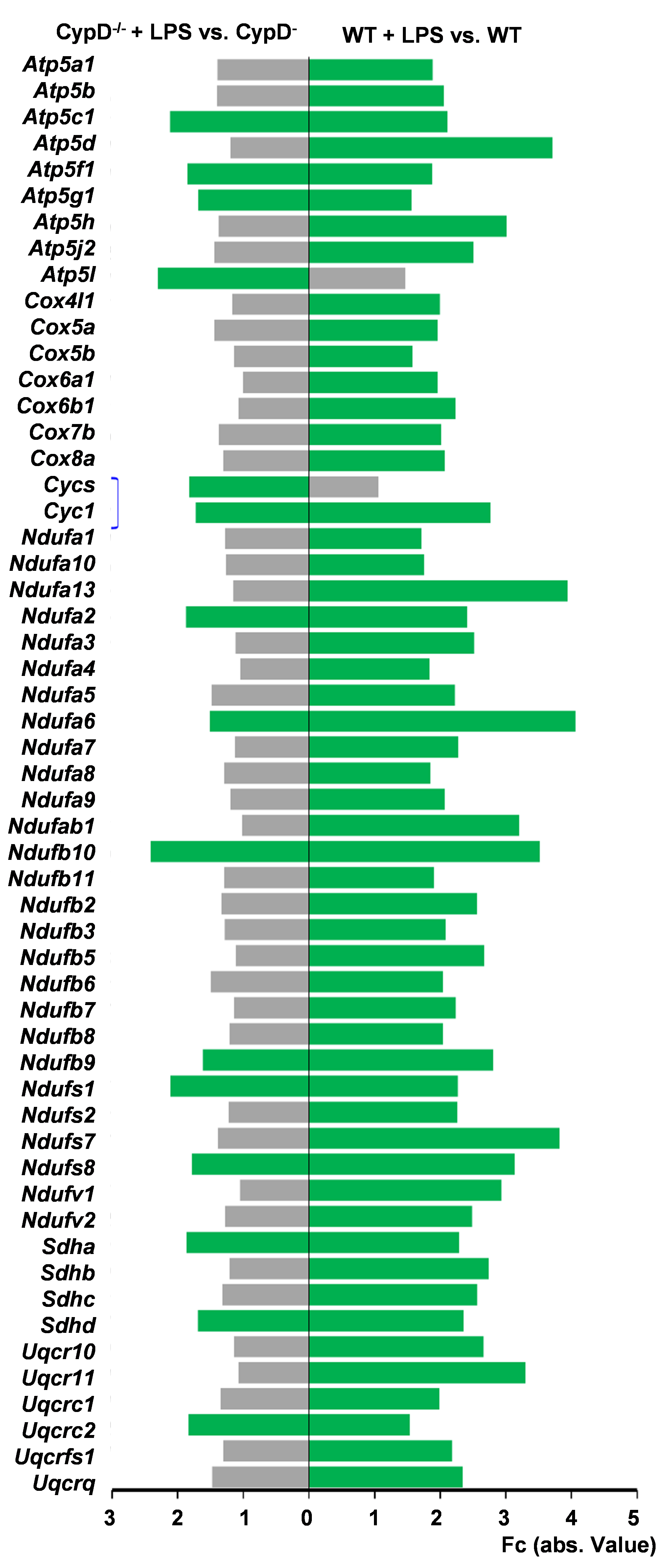

Supplement: Supplementary file 3 — Fig S3. Effect of CypD disruption on the LPS induced differential expression of nuclear DNA encoded genes of oxidative phosphorylation. [file FEB4-11-684-s003.tif]
