# Supplementary material for: Cyclophilin D‐dependent mitochondrial permeability transition amplifies inflammatory reprogramming in endotoxemia
Source: FEBS Open Bio. 2021 Feb 13;11(3):684–704. doi: 10.1002/2211-5463.13091 (PMC7931201; doi:10.1002/2211-5463.13091)
Supplement: Supplementary file 4 — Table S1. LPS induced significant enrichment of DEGs in WT mice liver tissue in canonical pathways analyzed by IPA. ‐lg(adjPval.) > 1.3. [file FEB4-11-684-s004.docx]

| **Ingenuity Canonical Pathways** | **-log(B-H p-value)** | **Ratio** | **z-score** | **Downregulated** | **No change** | **Upregulated** | **No overlap with dataset** |
| --- | --- | --- | --- | --- | --- | --- | --- |
| Mitochondrial Dysfunction | 26.70 | 46.8% | N/A | 76/171 (44%) | 0/171 (0%) | 4/171 (2%) | 91/171 (53%) |
| LPS/IL-1 Mediated Inhibition of RXR Function | 23.40 | 39.6% | 4.116 | 74/222 (33%) | 0/222 (0%) | 14/222 (6%) | 134/222 (60%) |
| Sirtuin Signaling Pathway | 23.10 | 35.3% | 1.51 | 80/292 (27%) | 0/292 (0%) | 23/292 (8%) | 189/292 (65%) |
| Oxidative Phosphorylation | 22.10 | 52.3% | -7.55 | 57/109 (52%) | 0/109 (0%) | 0/109 (0%) | 52/109 (48%) |
| FXR/RXR Activation | 15.90 | 42.9% | N/A | 42/126 (33%) | 0/126 (0%) | 12/126 (10%) | 72/126 (57%) |
| LXR/RXR Activation | 15.40 | 43.0% | -3.395 | 31/121 (26%) | 0/121 (0%) | 21/121 (17%) | 69/121 (57%) |
| PXR/RXR Activation | 12.80 | 52.3% | N/A | 32/65 (49%) | 0/65 (0%) | 2/65 (3%) | 31/65 (48%) |
| Xenobiotic Metabolism Signaling | 11.50 | 27.9% | N/A | 67/290 (23%) | 0/290 (0%) | 14/290 (5%) | 209/290 (72%) |
| Acute Phase Response Signaling | 10.80 | 32.9% | 2.967 | 19/170 (11%) | 0/170 (0%) | 37/170 (22%) | 114/170 (67%) |
| Superpathway of Cholesterol Biosynthesis | 10.80 | 71.4% | -4.472 | 20/28 (71%) | 0/28 (0%) | 0/28 (0%) | 8/28 (29%) |
| Stearate Biosynthesis I (Animals) | 9.40 | 54.5% | -4.082 | 22/44 (50%) | 0/44 (0%) | 2/44 (5%) | 20/44 (45%) |
| Serotonin Degradation | 8.82 | 41.6% | -4.95 | 30/77 (39%) | 0/77 (0%) | 2/77 (3%) | 45/77 (58%) |
| Nicotine Degradation II | 8.82 | 44.6% | -5.014 | 28/65 (43%) | 0/65 (0%) | 1/65 (2%) | 36/65 (55%) |
| Aryl Hydrocarbon Receptor Signaling | 8.72 | 32.6% | -0.688 | 33/141 (23%) | 0/141 (0%) | 13/141 (9%) | 95/141 (67%) |
| Tryptophan Degradation III (Eukaryotic) | 8.59 | 68.0% | -4.123 | 17/25 (68%) | 0/25 (0%) | 0/25 (0%) | 8/25 (32%) |
| Fatty Acid β-oxidation I | 8.23 | 59.4% | -4.359 | 19/32 (59%) | 0/32 (0%) | 0/32 (0%) | 13/32 (41%) |
| Glutathione-mediated Detoxification | 7.55 | 58.1% | -4.243 | 18/31 (58%) | 0/31 (0%) | 0/31 (0%) | 13/31 (42%) |
| Nicotine Degradation III | 6.86 | 42.9% | -4.491 | 23/56 (41%) | 0/56 (0%) | 1/56 (2%) | 32/56 (57%) |
| Ethanol Degradation II | 6.86 | 51.4% | -3.9 | 18/37 (49%) | 0/37 (0%) | 1/37 (3%) | 18/37 (49%) |
| Hepatic Cholestasis | 6.86 | 28.8% | N/A | 29/160 (18%) | 0/160 (0%) | 17/160 (11%) | 114/160 (71%) |
| TCA Cycle II (Eukaryotic) | 6.83 | 62.5% | -3.873 | 15/24 (63%) | 0/24 (0%) | 0/24 (0%) | 9/24 (38%) |
| NRF2-mediated Oxidative Stress Response | 6.82 | 26.9% | -1.569 | 37/193 (19%) | 0/193 (0%) | 15/193 (8%) | 141/193 (73%) |
| Melatonin Degradation I | 6.81 | 40.0% | -4.707 | 25/65 (38%) | 0/65 (0%) | 1/65 (2%) | 39/65 (60%) |
| Superpathway of Melatonin Degradation | 6.71 | 38.6% | -4.811 | 26/70 (37%) | 0/70 (0%) | 1/70 (1%) | 43/70 (61%) |
| Glycine Betaine Degradation | 6.03 | 90.0% | -2.333 | 8/10 (80%) | 0/10 (0%) | 1/10 (10%) | 1/10 (10%) |
| Glutathione Redox Reactions I | 5.87 | 58.3% | -3.207 | 13/24 (54%) | 0/24 (0%) | 1/24 (4%) | 10/24 (42%) |
| Mevalonate Pathway I | 5.65 | 76.9% | -3.162 | 10/13 (77%) | 0/13 (0%) | 0/13 (0%) | 3/13 (23%) |
| Bile Acid Biosynthesis, Neutral Pathway | 5.65 | 76.9% | -3.162 | 10/13 (77%) | 0/13 (0%) | 0/13 (0%) | 3/13 (23%) |
| Glutaryl-CoA Degradation | 5.51 | 68.8% | -3.317 | 11/16 (69%) | 0/16 (0%) | 0/16 (0%) | 5/16 (31%) |
| Estrogen Biosynthesis | 5.31 | 43.9% | -4.243 | 18/41 (44%) | 0/41 (0%) | 0/41 (0%) | 23/41 (56%) |
| Acetone Degradation I (to Methylglyoxal) | 5.23 | 50.0% | -3.873 | 15/30 (50%) | 0/30 (0%) | 0/30 (0%) | 15/30 (50%) |
| Superpathway of Geranylgeranyldiphosphate Biosynthesis I (via Mevalonate) | 5.15 | 64.7% | -3.317 | 11/17 (65%) | 0/17 (0%) | 0/17 (0%) | 6/17 (35%) |
| Coagulation System | 4.98 | 45.7% | 0 | 9/35 (26%) | 0/35 (0%) | 7/35 (20%) | 19/35 (54%) |
| Noradrenaline and Adrenaline Degradation | 4.79 | 42.5% | -3.638 | 16/40 (40%) | 0/40 (0%) | 1/40 (3%) | 23/40 (57%) |
| Ketogenesis | 4.64 | 80.0% | -2.828 | 8/10 (80%) | 0/10 (0%) | 0/10 (0%) | 2/10 (20%) |
| Cholesterol Biosynthesis I | 4.50 | 69.2% | -3 | 9/13 (69%) | 0/13 (0%) | 0/13 (0%) | 4/13 (31%) |
| Cholesterol Biosynthesis II (via 24,25-dihydrolanosterol) | 4.50 | 69.2% | -3 | 9/13 (69%) | 0/13 (0%) | 0/13 (0%) | 4/13 (31%) |
| Cholesterol Biosynthesis III (via Desmosterol) | 4.50 | 69.2% | -3 | 9/13 (69%) | 0/13 (0%) | 0/13 (0%) | 4/13 (31%) |
| Extrinsic Prothrombin Activation Pathway | 4.49 | 62.5% | -1 | 6/16 (38%) | 0/16 (0%) | 4/16 (25%) | 6/16 (38%) |
| Iron homeostasis signaling pathway | 4.35 | 26.3% | N/A | 22/133 (17%) | 0/133 (0%) | 13/133 (10%) | 98/133 (74%) |
| PPARα/RXRα Activation | 4.25 | 23.9% | -2.967 | 25/180 (14%) | 0/180 (0%) | 18/180 (10%) | 137/180 (76%) |
| Triacylglycerol Degradation | 4.18 | 35.8% | -3.9 | 18/53 (34%) | 0/53 (0%) | 1/53 (2%) | 34/53 (64%) |
| Oxidative Ethanol Degradation III | 4.02 | 52.4% | -3.317 | 11/21 (52%) | 0/21 (0%) | 0/21 (0%) | 10/21 (48%) |
| Ethanol Degradation IV | 3.95 | 48.0% | -3.464 | 12/25 (48%) | 0/25 (0%) | 0/25 (0%) | 13/25 (52%) |
| Valine Degradation I | 3.92 | 55.6% | -3.162 | 10/18 (56%) | 0/18 (0%) | 0/18 (0%) | 8/18 (44%) |
| Heme Biosynthesis II | 3.92 | 77.8% | -2.646 | 7/9 (78%) | 0/9 (0%) | 0/9 (0%) | 2/9 (22%) |
| Acyl-CoA Hydrolysis | 3.82 | 66.7% | -1.414 | 6/12 (50%) | 0/12 (0%) | 2/12 (17%) | 4/12 (33%) |
| Production of Nitric Oxide and Reactive Oxygen Species in Macrophages | 3.81 | 22.7% | 0.762 | 21/194 (11%) | 0/194 (0%) | 23/194 (12%) | 150/194 (77%) |
| Toll-like Receptor Signaling | 3.81 | 30.3% | 2.138 | 5/76 (7%) | 0/76 (0%) | 18/76 (24%) | 53/76 (70%) |
| Thyroid Hormone Metabolism II (via Conjugation and/or Degradation) | 3.74 | 37.2% | -3.5 | 15/43 (35%) | 0/43 (0%) | 1/43 (2%) | 27/43 (63%) |
| Ubiquinol-10 Biosynthesis (Eukaryotic) | 3.69 | 52.6% | -3.162 | 10/19 (53%) | 0/19 (0%) | 0/19 (0%) | 9/19 (47%) |
| Putrescine Degradation III | 3.62 | 47.8% | -2.714 | 10/23 (43%) | 0/23 (0%) | 1/23 (4%) | 12/23 (52%) |
| Tetrapyrrole Biosynthesis II | 3.58 | 100.0% | -2.236 | 5/5 (100%) | 0/5 (0%) | 0/5 (0%) | 0/5 (0%) |
| Unfolded protein response | 3.42 | 32.7% | N/A | 8/55 (15%) | 0/55 (0%) | 10/55 (18%) | 37/55 (67%) |
| Dopamine Degradation | 3.34 | 37.8% | -3.207 | 13/37 (35%) | 0/37 (0%) | 1/37 (3%) | 23/37 (62%) |
| γ-linolenate Biosynthesis II (Animals) | 3.33 | 52.9% | -3 | 9/17 (53%) | 0/17 (0%) | 0/17 (0%) | 8/17 (47%) |
| Bupropion Degradation | 3.25 | 44.0% | -3.317 | 11/25 (44%) | 0/25 (0%) | 0/25 (0%) | 14/25 (56%) |
| Tryptophan Degradation X (Mammalian, via Tryptamine) | 3.25 | 44.0% | -3.317 | 11/25 (44%) | 0/25 (0%) | 0/25 (0%) | 14/25 (56%) |
| Isoleucine Degradation I | 3.23 | 57.1% | -2.828 | 8/14 (57%) | 0/14 (0%) | 0/14 (0%) | 6/14 (43%) |
| EIF2 Signaling | 3.11 | 20.8% | -1.768 | 27/221 (12%) | 0/221 (0%) | 19/221 (9%) | 175/221 (79%) |
| TNFR2 Signaling | 3.11 | 40.0% | 1.508 | 1/30 (3%) | 0/30 (0%) | 11/30 (37%) | 18/30 (60%) |
| RAR Activation | 3.11 | 21.6% | N/A | 28/190 (15%) | 0/190 (0%) | 13/190 (7%) | 149/190 (78%) |
| Superpathway of Citrulline Metabolism | 2.98 | 53.3% | -1.414 | 6/15 (40%) | 0/15 (0%) | 2/15 (13%) | 7/15 (47%) |
| Histidine Degradation VI | 2.98 | 53.3% | -2.828 | 8/15 (53%) | 0/15 (0%) | 0/15 (0%) | 7/15 (47%) |
| Histamine Degradation | 2.91 | 47.4% | -3 | 9/19 (47%) | 0/19 (0%) | 0/19 (0%) | 10/19 (53%) |
| Clathrin-mediated Endocytosis Signaling | 2.90 | 20.8% | N/A | 26/207 (13%) | 0/207 (0%) | 17/207 (8%) | 164/207 (79%) |
| Glucocorticoid Receptor Signaling | 2.87 | 18.6% | N/A | 20/339 (6%) | 0/339 (0%) | 43/339 (13%) | 276/339 (81%) |
| Triacylglycerol Biosynthesis | 2.85 | 32.6% | -2.84 | 13/46 (28%) | 0/46 (0%) | 2/46 (4%) | 31/46 (67%) |
| Phosphatidylethanolamine Biosynthesis II | 2.85 | 66.7% | -0.816 | 4/9 (44%) | 0/9 (0%) | 2/9 (22%) | 3/9 (33%) |
| Leucine Degradation I | 2.85 | 66.7% | -2.449 | 6/9 (67%) | 0/9 (0%) | 0/9 (0%) | 3/9 (33%) |
| Superpathway of Methionine Degradation | 2.80 | 35.1% | -2.496 | 11/37 (30%) | 0/37 (0%) | 2/37 (5%) | 24/37 (65%) |
| Methylmalonyl Pathway | 2.79 | 100.0% | -2 | 4/4 (100%) | 0/4 (0%) | 0/4 (0%) | 0/4 (0%) |
| Phenylalanine Degradation I (Aerobic) | 2.79 | 100.0% | -2 | 4/4 (100%) | 0/4 (0%) | 0/4 (0%) | 0/4 (0%) |
| PPAR Signaling | 2.78 | 25.3% | -2.041 | 7/95 (7%) | 0/95 (0%) | 17/95 (18%) | 71/95 (75%) |
| NF-κB Signaling | 2.68 | 21.0% | 0.822 | 11/181 (6%) | 0/181 (0%) | 27/181 (15%) | 143/181 (79%) |
| IL-10 Signaling | 2.65 | 27.5% | N/A | 3/69 (4%) | 0/69 (0%) | 16/69 (23%) | 50/69 (72%) |
| Oleate Biosynthesis II (Animals) | 2.65 | 53.8% | -2.646 | 7/13 (54%) | 0/13 (0%) | 0/13 (0%) | 6/13 (46%) |
| Atherosclerosis Signaling | 2.62 | 22.8% | N/A | 15/127 (12%) | 0/127 (0%) | 14/127 (11%) | 98/127 (77%) |
| Retinoate Biosynthesis I | 2.62 | 35.3% | -2.887 | 11/34 (32%) | 0/34 (0%) | 1/34 (3%) | 22/34 (65%) |
| TR/RXR Activation | 2.59 | 24.5% | N/A | 16/98 (16%) | 0/98 (0%) | 8/98 (8%) | 74/98 (76%) |
| Glycolysis I | 2.48 | 38.5% | -1.897 | 8/26 (31%) | 0/26 (0%) | 2/26 (8%) | 16/26 (62%) |
| Gluconeogenesis I | 2.48 | 38.5% | -2.53 | 9/26 (35%) | 0/26 (0%) | 1/26 (4%) | 16/26 (62%) |
| Androgen Biosynthesis | 2.44 | 50.0% | -2.646 | 7/14 (50%) | 0/14 (0%) | 0/14 (0%) | 7/14 (50%) |
| Phenylalanine Degradation IV (Mammalian, via Side Chain) | 2.44 | 50.0% | -1.89 | 6/14 (43%) | 0/14 (0%) | 1/14 (7%) | 7/14 (50%) |
| Polyamine Regulation in Colon Cancer | 2.44 | 40.9% | N/A | 5/22 (23%) | 0/22 (0%) | 4/22 (18%) | 13/22 (59%) |
| Fatty Acid α-oxidation | 2.44 | 40.9% | -3 | 9/22 (41%) | 0/22 (0%) | 0/22 (0%) | 13/22 (59%) |
| IL-12 Signaling and Production in Macrophages | 2.28 | 21.2% | N/A | 17/146 (12%) | 0/146 (0%) | 14/146 (10%) | 115/146 (79%) |
| Complement System | 2.28 | 32.4% | -2.333 | 12/37 (32%) | 0/37 (0%) | 0/37 (0%) | 25/37 (68%) |
| Remodeling of Epithelial Adherens Junctions | 2.26 | 26.1% | 0 | 10/69 (14%) | 0/69 (0%) | 8/69 (12%) | 51/69 (74%) |
| IL-1 Signaling | 2.25 | 23.9% | 0.655 | 8/92 (9%) | 0/92 (0%) | 14/92 (15%) | 70/92 (76%) |
| Methylglyoxal Degradation III | 2.25 | 42.1% | -2.828 | 8/19 (42%) | 0/19 (0%) | 0/19 (0%) | 11/19 (58%) |
| NAD biosynthesis II (from tryptophan) | 2.25 | 46.7% | -2.646 | 7/15 (47%) | 0/15 (0%) | 0/15 (0%) | 8/15 (53%) |
| 2-oxobutanoate Degradation I | 2.25 | 80.0% | -2 | 4/5 (80%) | 0/5 (0%) | 0/5 (0%) | 1/5 (20%) |
| Tyrosine Degradation I | 2.25 | 80.0% | -1 | 3/5 (60%) | 0/5 (0%) | 1/5 (20%) | 1/5 (20%) |
| Folate Polyglutamylation | 2.25 | 80.0% | -2 | 4/5 (80%) | 0/5 (0%) | 0/5 (0%) | 1/5 (20%) |
| Histidine Degradation III | 2.23 | 62.5% | -2.236 | 5/8 (63%) | 0/8 (0%) | 0/8 (0%) | 3/8 (38%) |
| Tryptophan Degradation to 2-amino-3-carboxymuconate Semialdehyde | 2.23 | 62.5% | -2.236 | 5/8 (63%) | 0/8 (0%) | 0/8 (0%) | 3/8 (38%) |
| Hepatic Fibrosis / Hepatic Stellate Cell Activation | 2.21 | 19.8% | N/A | 12/187 (6%) | 0/187 (0%) | 25/187 (13%) | 150/187 (80%) |
| PI3K/AKT Signaling | 2.13 | 21.6% | 0.577 | 11/125 (9%) | 0/125 (0%) | 16/125 (13%) | 98/125 (78%) |
| Dopamine Receptor Signaling | 2.13 | 24.7% | -0.378 | 15/77 (19%) | 0/77 (0%) | 4/77 (5%) | 58/77 (75%) |
| Glucocorticoid Biosynthesis | 2.10 | 50.0% | -2.449 | 6/12 (50%) | 0/12 (0%) | 0/12 (0%) | 6/12 (50%) |
| TNFR1 Signaling | 2.06 | 28.0% | 1.387 | 2/50 (4%) | 0/50 (0%) | 12/50 (24%) | 36/50 (72%) |
| Apoptosis Signaling | 2.05 | 23.3% | -0.447 | 8/90 (9%) | 0/90 (0%) | 13/90 (14%) | 69/90 (77%) |
| Tetrahydrobiopterin Biosynthesis I | 2.03 | 100.0% | N/A | 3/3 (100%) | 0/3 (0%) | 0/3 (0%) | 0/3 (0%) |
| D-glucuronate Degradation I | 2.03 | 100.0% | N/A | 3/3 (100%) | 0/3 (0%) | 0/3 (0%) | 0/3 (0%) |
| Tetrahydrobiopterin Biosynthesis II | 2.03 | 100.0% | N/A | 3/3 (100%) | 0/3 (0%) | 0/3 (0%) | 0/3 (0%) |
| Tyrosine Biosynthesis IV | 2.03 | 100.0% | N/A | 3/3 (100%) | 0/3 (0%) | 0/3 (0%) | 0/3 (0%) |
| TWEAK Signaling | 2.03 | 31.4% | 0.302 | 3/35 (9%) | 0/35 (0%) | 8/35 (23%) | 24/35 (69%) |
| IL-6 Signaling | 2.01 | 21.1% | 2.6 | 5/128 (4%) | 0/128 (0%) | 22/128 (17%) | 101/128 (79%) |
| Maturity Onset Diabetes of Young (MODY) Signaling | 2.00 | 38.1% | N/A | 8/21 (38%) | 0/21 (0%) | 0/21 (0%) | 13/21 (62%) |
| Citrulline Biosynthesis | 1.98 | 55.6% | -1.342 | 4/9 (44%) | 0/9 (0%) | 1/9 (11%) | 4/9 (44%) |
| Phagosome Maturation | 1.97 | 20.3% | N/A | 18/148 (12%) | 0/148 (0%) | 12/148 (8%) | 118/148 (80%) |
| Estrogen-Dependent Breast Cancer Signaling | 1.97 | 23.8% | 0.577 | 12/80 (15%) | 0/80 (0%) | 7/80 (9%) | 61/80 (76%) |
| Mitochondrial L-carnitine Shuttle Pathway | 1.95 | 41.2% | -2.646 | 7/17 (41%) | 0/17 (0%) | 0/17 (0%) | 10/17 (59%) |
| Role of IL-17A in Psoriasis | 1.94 | 46.2% | N/A | 1/13 (8%) | 0/13 (0%) | 5/13 (38%) | 7/13 (54%) |
| Fatty Acid Activation | 1.94 | 46.2% | -2.449 | 6/13 (46%) | 0/13 (0%) | 0/13 (0%) | 7/13 (54%) |
| Heparan Sulfate Biosynthesis (Late Stages) | 1.93 | 24.0% | -3.771 | 17/75 (23%) | 0/75 (0%) | 1/75 (1%) | 57/75 (76%) |
| Death Receptor Signaling | 1.92 | 22.6% | 0.894 | 5/93 (5%) | 0/93 (0%) | 16/93 (17%) | 72/93 (77%) |
| Arginine Biosynthesis IV | 1.92 | 66.7% | -2 | 4/6 (67%) | 0/6 (0%) | 0/6 (0%) | 2/6 (33%) |
| Zymosterol Biosynthesis | 1.92 | 66.7% | -2 | 4/6 (67%) | 0/6 (0%) | 0/6 (0%) | 2/6 (33%) |
| Intrinsic Prothrombin Activation Pathway | 1.89 | 28.6% | -0.632 | 8/42 (19%) | 0/42 (0%) | 4/42 (10%) | 30/42 (71%) |
| Retinol Biosynthesis | 1.89 | 28.6% | -2.887 | 11/42 (26%) | 0/42 (0%) | 1/42 (2%) | 30/42 (71%) |
| Heparan Sulfate Biosynthesis | 1.88 | 23.2% | -3.9 | 18/82 (22%) | 0/82 (0%) | 1/82 (1%) | 63/82 (77%) |
| Ketolysis | 1.77 | 50.0% | -2.236 | 5/10 (50%) | 0/10 (0%) | 0/10 (0%) | 5/10 (50%) |
| γ-glutamyl Cycle | 1.77 | 42.9% | -1.633 | 5/14 (36%) | 0/14 (0%) | 1/14 (7%) | 8/14 (57%) |
| Type II Diabetes Mellitus Signaling | 1.75 | 19.5% | 0.229 | 18/154 (12%) | 0/154 (0%) | 12/154 (8%) | 124/154 (81%) |
| Prostate Cancer Signaling | 1.73 | 21.6% | N/A | 9/97 (9%) | 0/97 (0%) | 12/97 (12%) | 76/97 (78%) |
| Salvage Pathways of Pyrimidine Ribonucleotides | 1.73 | 21.6% | -0.655 | 12/97 (12%) | 0/97 (0%) | 9/97 (9%) | 76/97 (78%) |
| STAT3 Pathway | 1.73 | 21.6% | -0.655 | 8/97 (8%) | 0/97 (0%) | 13/97 (13%) | 76/97 (78%) |
| tRNA Charging | 1.70 | 28.2% | -1.508 | 8/39 (21%) | 0/39 (0%) | 3/39 (8%) | 28/39 (72%) |
| Protein Ubiquitination Pathway | 1.69 | 17.4% | N/A | 21/265 (8%) | 0/265 (0%) | 25/265 (9%) | 219/265 (83%) |
| p53 Signaling | 1.67 | 20.7% | -0.447 | 9/111 (8%) | 0/111 (0%) | 14/111 (13%) | 88/111 (79%) |
| Tumoricidal Function of Hepatic Natural Killer Cells | 1.67 | 33.3% | 0 | 3/24 (13%) | 0/24 (0%) | 5/24 (21%) | 16/24 (67%) |
| CDP-diacylglycerol Biosynthesis I | 1.67 | 33.3% | -2.828 | 8/24 (33%) | 0/24 (0%) | 0/24 (0%) | 16/24 (67%) |
| Phosphatidylcholine Biosynthesis I | 1.64 | 57.1% | 0 | 2/7 (29%) | 0/7 (0%) | 2/7 (29%) | 3/7 (43%) |
| Aspartate Degradation II | 1.64 | 57.1% | -1 | 3/7 (43%) | 0/7 (0%) | 1/7 (14%) | 3/7 (43%) |
| Glycogen Degradation III | 1.64 | 40.0% | -1.633 | 5/15 (33%) | 0/15 (0%) | 1/15 (7%) | 9/15 (60%) |
| Acute Myeloid Leukemia Signaling | 1.64 | 21.5% | 0.775 | 12/93 (13%) | 0/93 (0%) | 8/93 (9%) | 73/93 (78%) |
| Osteoarthritis Pathway | 1.64 | 17.9% | 1.061 | 9/212 (4%) | 0/212 (0%) | 29/212 (14%) | 174/212 (82%) |
| Mineralocorticoid Biosynthesis | 1.61 | 45.5% | -2.236 | 5/11 (45%) | 0/11 (0%) | 0/11 (0%) | 6/11 (55%) |
| IL-17A Signaling in Fibroblasts | 1.61 | 28.6% | N/A | 2/35 (6%) | 0/35 (0%) | 8/35 (23%) | 25/35 (71%) |
| Arsenate Detoxification I (Glutaredoxin) | 1.61 | 75.0% | N/A | 2/4 (50%) | 0/4 (0%) | 1/4 (25%) | 1/4 (25%) |
| Uracil Degradation II (Reductive) | 1.61 | 75.0% | N/A | 3/4 (75%) | 0/4 (0%) | 0/4 (0%) | 1/4 (25%) |
| Spermine and Spermidine Degradation I | 1.61 | 75.0% | N/A | 2/4 (50%) | 0/4 (0%) | 1/4 (25%) | 1/4 (25%) |
| α-tocopherol Degradation | 1.61 | 75.0% | N/A | 3/4 (75%) | 0/4 (0%) | 0/4 (0%) | 1/4 (25%) |
| Glutathione Redox Reactions II | 1.61 | 75.0% | N/A | 0/4 (0%) | 0/4 (0%) | 3/4 (75%) | 1/4 (25%) |
| Thymine Degradation | 1.61 | 75.0% | N/A | 3/4 (75%) | 0/4 (0%) | 0/4 (0%) | 1/4 (25%) |
| Acetate Conversion to Acetyl-CoA | 1.61 | 75.0% | N/A | 3/4 (75%) | 0/4 (0%) | 0/4 (0%) | 1/4 (25%) |
| Epithelial Adherens Junction Signaling | 1.61 | 19.2% | N/A | 16/146 (11%) | 0/146 (0%) | 12/146 (8%) | 118/146 (81%) |
| IL-17A Signaling in Gastric Cells | 1.59 | 32.0% | 0.447 | 2/25 (8%) | 0/25 (0%) | 6/25 (24%) | 17/25 (68%) |
| JAK/Stat Signaling | 1.54 | 21.7% | 0 | 5/83 (6%) | 0/83 (0%) | 13/83 (16%) | 65/83 (78%) |
| AMPK Signaling | 1.54 | 17.6% | -1.134 | 27/216 (13%) | 0/216 (0%) | 11/216 (5%) | 178/216 (82%) |
| Chondroitin Sulfate Degradation (Metazoa) | 1.52 | 37.5% | -0.816 | 4/16 (25%) | 0/16 (0%) | 2/16 (13%) | 10/16 (63%) |
| Phosphatidylglycerol Biosynthesis II (Non-plastidic) | 1.49 | 30.8% | -2.828 | 8/26 (31%) | 0/26 (0%) | 0/26 (0%) | 18/26 (69%) |
| Pregnenolone Biosynthesis | 1.46 | 41.7% | -2.236 | 5/12 (42%) | 0/12 (0%) | 0/12 (0%) | 7/12 (58%) |
| Induction of Apoptosis by HIV1 | 1.40 | 23.0% | 0 | 3/61 (5%) | 0/61 (0%) | 11/61 (18%) | 47/61 (77%) |
| Dermatan Sulfate Degradation (Metazoa) | 1.39 | 35.3% | -0.816 | 4/17 (24%) | 0/17 (0%) | 2/17 (12%) | 11/17 (65%) |
| Antigen Presentation Pathway | 1.38 | 26.3% | N/A | 1/38 (3%) | 0/38 (0%) | 9/38 (24%) | 28/38 (74%) |
| IGF-1 Signaling | 1.37 | 19.8% | -1.5 | 11/106 (10%) | 0/106 (0%) | 10/106 (9%) | 85/106 (80%) |
| Glycogen Degradation II | 1.31 | 38.5% | -1.342 | 4/13 (31%) | 0/13 (0%) | 1/13 (8%) | 8/13 (62%) |
| Role of IL-17A in Arthritis | 1.31 | 21.7% | N/A | 4/69 (6%) | 0/69 (0%) | 11/69 (16%) | 54/69 (78%) |
| Insulin Receptor Signaling | 1.31 | 18.4% | 0 | 19/141 (13%) | 0/141 (0%) | 7/141 (5%) | 115/141 (82%) |
| Neuroinflammation Signaling Pathway | 1.31 | 16.1% | 2.592 | 20/311 (6%) | 0/311 (0%) | 30/311 (10%) | 261/311 (84%) |
| iNOS Signaling | 1.31 | 24.4% | 3.162 | 0/45 (0%) | 0/45 (0%) | 11/45 (24%) | 34/45 (76%) |
| Lysine Degradation V | 1.31 | 60.0% | N/A | 3/5 (60%) | 0/5 (0%) | 0/5 (0%) | 2/5 (40%) |
| Galactose Degradation I (Leloir Pathway) | 1.31 | 60.0% | N/A | 3/5 (60%) | 0/5 (0%) | 0/5 (0%) | 2/5 (40%) |
| dTMP De Novo Biosynthesis | 1.31 | 60.0% | N/A | 3/5 (60%) | 0/5 (0%) | 0/5 (0%) | 2/5 (40%) |
| ILK Signaling | 1.31 | 17.3% | 0.365 | 16/197 (8%) | 0/197 (0%) | 18/197 (9%) | 163/197 (83%) |
| Glycine Biosynthesis III | 1.31 | 100.0% | N/A | 2/2 (100%) | 0/2 (0%) | 0/2 (0%) | 0/2 (0%) |
| Choline Degradation I | 1.31 | 100.0% | N/A | 2/2 (100%) | 0/2 (0%) | 0/2 (0%) | 0/2 (0%) |
| L-cysteine Degradation III | 1.31 | 100.0% | N/A | 1/2 (50%) | 0/2 (0%) | 1/2 (50%) | 0/2 (0%) |
| Glycine Degradation (Creatine Biosynthesis) | 1.31 | 100.0% | N/A | 2/2 (100%) | 0/2 (0%) | 0/2 (0%) | 0/2 (0%) |
| Cysteine Biosynthesis/Homocysteine Degradation | 1.31 | 100.0% | N/A | 2/2 (100%) | 0/2 (0%) | 0/2 (0%) | 0/2 (0%) |
| Adenine and Adenosine Salvage I | 1.31 | 100.0% | N/A | 1/2 (50%) | 0/2 (0%) | 1/2 (50%) | 0/2 (0%) |
| Formaldehyde Oxidation II (Glutathione-dependent) | 1.31 | 100.0% | N/A | 2/2 (100%) | 0/2 (0%) | 0/2 (0%) | 0/2 (0%) |
| Glycine Biosynthesis I | 1.31 | 100.0% | N/A | 2/2 (100%) | 0/2 (0%) | 0/2 (0%) | 0/2 (0%) |
| Glutamine Degradation I | 1.31 | 100.0% | N/A | 1/2 (50%) | 0/2 (0%) | 1/2 (50%) | 0/2 (0%) |
| DNA Methylation and Transcriptional Repression Signaling | 1.30 | 26.5% | N/A | 8/34 (24%) | 0/34 (0%) | 1/34 (3%) | 25/34 (74%) |
