# Supplementary material for: Cyclophilin D‐dependent mitochondrial permeability transition amplifies inflammatory reprogramming in endotoxemia
Source: FEBS Open Bio. 2021 Feb 13;11(3):684–704. doi: 10.1002/2211-5463.13091 (PMC7931201; doi:10.1002/2211-5463.13091)
Supplement: Supplementary file 5 — Table S2. LPS induced significant enrichment of DEGs in CypD‐/‐ mice liver tissue in canonical pathways analyzed by IPA. ‐lg(adjPval.) > 1.3. [file FEB4-11-684-s005.docx]

| **Ingenuity Canonical Pathways** | **-log(B-H p-value)** | **Ratio** | **z-score** | **Downregulated** | **No change** | **Upregulated** | **No overlap with dataset** |
| --- | --- | --- | --- | --- | --- | --- | --- |
| LXR/RXR Activation | 23.80 | 39.7% | -2.333 | 25/121 (21%) | 0/121 (0%) | 23/121 (19%) | 73/121 (60%) |
| LPS/IL-1 Mediated Inhibition of RXR Function | 23.50 | 28.8% | 2.694 | 48/222 (22%) | 0/222 (0%) | 16/222 (7%) | 158/222 (71%) |
| FXR/RXR Activation | 20.40 | 35.7% | N/A | 30/126 (24%) | 0/126 (0%) | 15/126 (12%) | 81/126 (64%) |
| Acute Phase Response Signaling | 13.90 | 25.9% | 3.43 | 8/170 (5%) | 0/170 (0%) | 36/170 (21%) | 126/170 (74%) |
| Fatty Acid β-oxidation I | 12.90 | 59.4% | -3.9 | 18/32 (56%) | 0/32 (0%) | 1/32 (3%) | 13/32 (41%) |
| Nicotine Degradation II | 12.80 | 40.0% | -4.315 | 24/65 (37%) | 0/65 (0%) | 2/65 (3%) | 39/65 (60%) |
| Superpathway of Cholesterol Biosynthesis | 11.80 | 60.7% | -4.123 | 17/28 (61%) | 0/28 (0%) | 0/28 (0%) | 11/28 (39%) |
| Nicotine Degradation III | 11.60 | 41.1% | -3.962 | 21/56 (38%) | 0/56 (0%) | 2/56 (4%) | 33/56 (59%) |
| PXR/RXR Activation | 11.00 | 36.9% | N/A | 20/65 (31%) | 0/65 (0%) | 4/65 (6%) | 41/65 (63%) |
| Sirtuin Signaling Pathway | 10.50 | 18.2% | -0.156 | 35/292 (12%) | 0/292 (0%) | 18/292 (6%) | 239/292 (82%) |
| Superpathway of Melatonin Degradation | 10.20 | 34.3% | -4.082 | 22/70 (31%) | 0/70 (0%) | 2/70 (3%) | 46/70 (66%) |
| Xenobiotic Metabolism Signaling | 10.20 | 17.9% | N/A | 42/290 (14%) | 0/290 (0%) | 10/290 (3%) | 238/290 (82%) |
| Melatonin Degradation I | 10.20 | 35.4% | -3.962 | 21/65 (32%) | 0/65 (0%) | 2/65 (3%) | 42/65 (65%) |
| Tryptophan Degradation III (Eukaryotic) | 9.10 | 56.0% | -3.742 | 14/25 (56%) | 0/25 (0%) | 0/25 (0%) | 11/25 (44%) |
| Serotonin Degradation | 8.52 | 29.9% | -3.962 | 21/77 (27%) | 0/77 (0%) | 2/77 (3%) | 54/77 (70%) |
| Hepatic Cholestasis | 8.47 | 21.2% | N/A | 20/160 (13%) | 0/160 (0%) | 14/160 (9%) | 126/160 (79%) |
| Glycine Betaine Degradation | 8.47 | 90.0% | -1.667 | 7/10 (70%) | 0/10 (0%) | 2/10 (20%) | 1/10 (10%) |
| Acetone Degradation I (to Methylglyoxal) | 7.82 | 46.7% | -2.673 | 12/30 (40%) | 0/30 (0%) | 2/30 (7%) | 16/30 (53%) |
| Glutaryl-CoA Degradation | 6.96 | 62.5% | -3.162 | 10/16 (63%) | 0/16 (0%) | 0/16 (0%) | 6/16 (38%) |
| Coagulation System | 6.79 | 40.0% | 0 | 8/35 (23%) | 0/35 (0%) | 6/35 (17%) | 21/35 (60%) |
| Estrogen Biosynthesis | 6.73 | 36.6% | -3.357 | 14/41 (34%) | 0/41 (0%) | 1/41 (2%) | 26/41 (63%) |
| Stearate Biosynthesis I (Animals) | 6.26 | 34.1% | -3.357 | 14/44 (32%) | 0/44 (0%) | 1/44 (2%) | 29/44 (66%) |
| Bupropion Degradation | 5.72 | 44.0% | -2.714 | 10/25 (40%) | 0/25 (0%) | 1/25 (4%) | 14/25 (56%) |
| Ethanol Degradation II | 5.57 | 35.1% | -3.051 | 12/37 (32%) | 0/37 (0%) | 1/37 (3%) | 24/37 (65%) |
| Superpathway of Methionine Degradation | 5.57 | 35.1% | -2.496 | 11/37 (30%) | 0/37 (0%) | 2/37 (5%) | 24/37 (65%) |
| Mitochondrial Dysfunction | 5.57 | 17.5% | N/A | 27/171 (16%) | 0/171 (0%) | 3/171 (2%) | 141/171 (82%) |
| Superpathway of Geranylgeranyldiphosphate Biosynthesis I (via Mevalonate) | 5.51 | 52.9% | -3 | 9/17 (53%) | 0/17 (0%) | 0/17 (0%) | 8/17 (47%) |
| Cholesterol Biosynthesis I | 5.51 | 61.5% | -2.828 | 8/13 (62%) | 0/13 (0%) | 0/13 (0%) | 5/13 (38%) |
| Mevalonate Pathway I | 5.51 | 61.5% | -2.828 | 8/13 (62%) | 0/13 (0%) | 0/13 (0%) | 5/13 (38%) |
| Bile Acid Biosynthesis, Neutral Pathway | 5.51 | 61.5% | -2.828 | 8/13 (62%) | 0/13 (0%) | 0/13 (0%) | 5/13 (38%) |
| Cholesterol Biosynthesis II (via 24,25-dihydrolanosterol) | 5.51 | 61.5% | -2.828 | 8/13 (62%) | 0/13 (0%) | 0/13 (0%) | 5/13 (38%) |
| Cholesterol Biosynthesis III (via Desmosterol) | 5.51 | 61.5% | -2.828 | 8/13 (62%) | 0/13 (0%) | 0/13 (0%) | 5/13 (38%) |
| NRF2-mediated Oxidative Stress Response | 5.50 | 16.6% | 0.243 | 17/193 (9%) | 0/193 (0%) | 15/193 (8%) | 161/193 (83%) |
| Valine Degradation I | 5.28 | 50.0% | -2.333 | 8/18 (44%) | 0/18 (0%) | 1/18 (6%) | 9/18 (50%) |
| Isoleucine Degradation I | 5.21 | 57.1% | -2.121 | 7/14 (50%) | 0/14 (0%) | 1/14 (7%) | 6/14 (43%) |
| TCA Cycle II (Eukaryotic) | 5.05 | 41.7% | -3.162 | 10/24 (42%) | 0/24 (0%) | 0/24 (0%) | 14/24 (58%) |
| Histidine Degradation VI | 4.92 | 53.3% | -2.121 | 7/15 (47%) | 0/15 (0%) | 1/15 (7%) | 7/15 (47%) |
| Thyroid Hormone Metabolism II (via Conjugation and/or Degradation) | 4.87 | 30.2% | -3.051 | 12/43 (28%) | 0/43 (0%) | 1/43 (2%) | 30/43 (70%) |
| Aryl Hydrocarbon Receptor Signaling | 4.79 | 17.7% | -1.265 | 18/141 (13%) | 0/141 (0%) | 7/141 (5%) | 116/141 (82%) |
| TR/RXR Activation | 4.71 | 20.4% | N/A | 13/98 (13%) | 0/98 (0%) | 7/98 (7%) | 78/98 (80%) |
| Production of Nitric Oxide and Reactive Oxygen Species in Macrophages | 4.56 | 15.5% | 1.134 | 13/194 (7%) | 0/194 (0%) | 17/194 (9%) | 164/194 (85%) |
| γ-linolenate Biosynthesis II (Animals) | 4.45 | 47.1% | -2.828 | 8/17 (47%) | 0/17 (0%) | 0/17 (0%) | 9/17 (53%) |
| Retinoate Biosynthesis I | 4.40 | 32.4% | -2.714 | 10/34 (29%) | 0/34 (0%) | 1/34 (3%) | 23/34 (68%) |
| Hepatic Fibrosis / Hepatic Stellate Cell Activation | 3.98 | 15.0% | N/A | 9/187 (5%) | 0/187 (0%) | 19/187 (10%) | 159/187 (85%) |
| Ketogenesis | 3.98 | 60.0% | -2.449 | 6/10 (60%) | 0/10 (0%) | 0/10 (0%) | 4/10 (40%) |
| Glutathione-mediated Detoxification | 3.97 | 32.3% | -3.162 | 10/31 (32%) | 0/31 (0%) | 0/31 (0%) | 21/31 (68%) |
| Ethanol Degradation IV | 3.97 | 36.0% | -3 | 9/25 (36%) | 0/25 (0%) | 0/25 (0%) | 16/25 (64%) |
| Triacylglycerol Degradation | 3.85 | 24.5% | -3.051 | 12/53 (23%) | 0/53 (0%) | 1/53 (2%) | 40/53 (75%) |
| IL-10 Signaling | 3.84 | 21.7% | N/A | 1/69 (1%) | 0/69 (0%) | 14/69 (20%) | 54/69 (78%) |
| Methylmalonyl Pathway | 3.81 | 100.0% | -2 | 4/4 (100%) | 0/4 (0%) | 0/4 (0%) | 0/4 (0%) |
| Noradrenaline and Adrenaline Degradation | 3.71 | 27.5% | -2.714 | 10/40 (25%) | 0/40 (0%) | 1/40 (3%) | 29/40 (73%) |
| Oxidative Ethanol Degradation III | 3.71 | 38.1% | -2.828 | 8/21 (38%) | 0/21 (0%) | 0/21 (0%) | 13/21 (62%) |
| Extrinsic Prothrombin Activation Pathway | 3.65 | 43.8% | N/A | 4/16 (25%) | 0/16 (0%) | 3/16 (19%) | 9/16 (56%) |
| IL-6 Signaling | 3.55 | 16.4% | 2.982 | 3/128 (2%) | 0/128 (0%) | 18/128 (14%) | 107/128 (84%) |
| Intrinsic Prothrombin Activation Pathway | 3.53 | 26.2% | -0.632 | 8/42 (19%) | 0/42 (0%) | 3/42 (7%) | 31/42 (74%) |
| RAR Activation | 3.51 | 14.2% | N/A | 17/190 (9%) | 0/190 (0%) | 10/190 (5%) | 163/190 (86%) |
| Granulocyte Adhesion and Diapedesis | 3.45 | 14.4% | N/A | 9/181 (5%) | 0/181 (0%) | 17/181 (9%) | 155/181 (86%) |
| Histidine Degradation III | 3.42 | 62.5% | -2.236 | 5/8 (63%) | 0/8 (0%) | 0/8 (0%) | 3/8 (38%) |
| Toll-like Receptor Signaling | 3.39 | 19.7% | 1.508 | 3/76 (4%) | 0/76 (0%) | 12/76 (16%) | 61/76 (80%) |
| TNFR2 Signaling | 3.35 | 30.0% | 2.121 | 1/30 (3%) | 0/30 (0%) | 8/30 (27%) | 21/30 (70%) |
| Complement System | 3.34 | 27.0% | 0 | 7/37 (19%) | 0/37 (0%) | 3/37 (8%) | 27/37 (73%) |
| Oleate Biosynthesis II (Animals) | 3.27 | 46.2% | -2.449 | 6/13 (46%) | 0/13 (0%) | 0/13 (0%) | 7/13 (54%) |
| Induction of Apoptosis by HIV1 | 3.27 | 21.3% | -0.277 | 4/61 (7%) | 0/61 (0%) | 9/61 (15%) | 48/61 (79%) |
| 2-oxobutanoate Degradation I | 3.25 | 80.0% | -2 | 4/5 (80%) | 0/5 (0%) | 0/5 (0%) | 1/5 (20%) |
| Tyrosine Degradation I | 3.25 | 80.0% | -1 | 3/5 (60%) | 0/5 (0%) | 1/5 (20%) | 1/5 (20%) |
| EIF2 Signaling | 3.22 | 13.1% | 0.728 | 8/221 (4%) | 0/221 (0%) | 21/221 (10%) | 192/221 (87%) |
| Ubiquinol-10 Biosynthesis (Eukaryotic) | 3.18 | 36.8% | -1.89 | 6/19 (32%) | 0/19 (0%) | 1/19 (5%) | 12/19 (63%) |
| Oxidative Phosphorylation | 3.12 | 16.5% | -4.243 | 18/109 (17%) | 0/109 (0%) | 0/109 (0%) | 91/109 (83%) |
| Phenylalanine Degradation IV (Mammalian, via Side Chain) | 3.10 | 42.9% | -1.633 | 5/14 (36%) | 0/14 (0%) | 1/14 (7%) | 8/14 (57%) |
| Clathrin-mediated Endocytosis Signaling | 2.95 | 13.0% | N/A | 17/207 (8%) | 0/207 (0%) | 10/207 (5%) | 180/207 (87%) |
| Iron homeostasis signaling pathway | 2.93 | 15.0% | N/A | 10/133 (8%) | 0/133 (0%) | 10/133 (8%) | 113/133 (85%) |
| Superpathway of Citrulline Metabolism | 2.92 | 40.0% | -1.633 | 5/15 (33%) | 0/15 (0%) | 1/15 (7%) | 9/15 (60%) |
| Zymosterol Biosynthesis | 2.85 | 66.7% | -2 | 4/6 (67%) | 0/6 (0%) | 0/6 (0%) | 2/6 (33%) |
| Interferon Signaling | 2.76 | 25.0% | 2.333 | 0/36 (0%) | 0/36 (0%) | 9/36 (25%) | 27/36 (75%) |
| Dopamine Degradation | 2.67 | 24.3% | -2.333 | 8/37 (22%) | 0/37 (0%) | 1/37 (3%) | 28/37 (76%) |
| Putrescine Degradation III | 2.64 | 30.4% | -1.89 | 6/23 (26%) | 0/23 (0%) | 1/23 (4%) | 16/23 (70%) |
| Type I Diabetes Mellitus Signaling | 2.58 | 15.3% | 1.387 | 3/111 (3%) | 0/111 (0%) | 14/111 (13%) | 94/111 (85%) |
| Unfolded protein response | 2.55 | 20.0% | N/A | 6/55 (11%) | 0/55 (0%) | 5/55 (9%) | 44/55 (80%) |
| Type II Diabetes Mellitus Signaling | 2.54 | 13.6% | 0 | 11/154 (7%) | 0/154 (0%) | 10/154 (6%) | 133/154 (86%) |
| Glutathione Redox Reactions I | 2.54 | 29.2% | -1.134 | 5/24 (21%) | 0/24 (0%) | 2/24 (8%) | 17/24 (71%) |
| Pregnenolone Biosynthesis | 2.50 | 41.7% | -1.342 | 4/12 (33%) | 0/12 (0%) | 1/12 (8%) | 7/12 (58%) |
| PPAR Signaling | 2.41 | 15.8% | -2.324 | 3/95 (3%) | 0/95 (0%) | 12/95 (13%) | 80/95 (84%) |
| Methylglyoxal Degradation III | 2.34 | 31.6% | -1.633 | 5/19 (26%) | 0/19 (0%) | 1/19 (5%) | 13/19 (68%) |
| Histamine Degradation | 2.34 | 31.6% | -2.449 | 6/19 (32%) | 0/19 (0%) | 0/19 (0%) | 13/19 (68%) |
| Fatty Acid Activation | 2.34 | 38.5% | -2.236 | 5/13 (38%) | 0/13 (0%) | 0/13 (0%) | 8/13 (62%) |
| Glycolysis I | 2.34 | 26.9% | -1.134 | 5/26 (19%) | 0/26 (0%) | 2/26 (8%) | 19/26 (73%) |
| Gluconeogenesis I | 2.34 | 26.9% | -1.89 | 6/26 (23%) | 0/26 (0%) | 1/26 (4%) | 19/26 (73%) |
| Protein Ubiquitination Pathway | 2.33 | 11.3% | N/A | 14/265 (5%) | 0/265 (0%) | 16/265 (6%) | 235/265 (89%) |
| Glucocorticoid Receptor Signaling | 2.32 | 10.6% | N/A | 7/339 (2%) | 0/339 (0%) | 29/339 (9%) | 303/339 (89%) |
| Retinol Biosynthesis | 2.31 | 21.4% | -2.333 | 8/42 (19%) | 0/42 (0%) | 1/42 (2%) | 33/42 (79%) |
| Tryptophan Degradation to 2-amino-3-carboxymuconate Semialdehyde | 2.31 | 50.0% | -2 | 4/8 (50%) | 0/8 (0%) | 0/8 (0%) | 4/8 (50%) |
| Phenylalanine Degradation I (Aerobic) | 2.28 | 75.0% | N/A | 3/4 (75%) | 0/4 (0%) | 0/4 (0%) | 1/4 (25%) |
| Acetate Conversion to Acetyl-CoA | 2.28 | 75.0% | N/A | 3/4 (75%) | 0/4 (0%) | 0/4 (0%) | 1/4 (25%) |
| Androgen Biosynthesis | 2.21 | 35.7% | -2.236 | 5/14 (36%) | 0/14 (0%) | 0/14 (0%) | 9/14 (64%) |
| γ-glutamyl Cycle | 2.21 | 35.7% | -1.342 | 4/14 (29%) | 0/14 (0%) | 1/14 (7%) | 9/14 (64%) |
| p53 Signaling | 2.21 | 14.4% | 0 | 6/111 (5%) | 0/111 (0%) | 10/111 (9%) | 95/111 (86%) |
| mTOR Signaling | 2.18 | 11.9% | 0.277 | 9/201 (4%) | 0/201 (0%) | 15/201 (7%) | 177/201 (88%) |
| Regulation of eIF4 and p70S6K Signaling | 2.13 | 12.7% | -0.816 | 7/157 (4%) | 0/157 (0%) | 13/157 (8%) | 137/157 (87%) |
| iNOS Signaling | 2.13 | 20.0% | 2.828 | 0/45 (0%) | 0/45 (0%) | 9/45 (20%) | 36/45 (80%) |
| IL-12 Signaling and Production in Macrophages | 2.13 | 13.0% | N/A | 8/146 (5%) | 0/146 (0%) | 11/146 (8%) | 127/146 (87%) |
| Citrulline Biosynthesis | 2.13 | 44.4% | -2 | 4/9 (44%) | 0/9 (0%) | 0/9 (0%) | 5/9 (56%) |
| Heme Biosynthesis II | 2.13 | 44.4% | -2 | 4/9 (44%) | 0/9 (0%) | 0/9 (0%) | 5/9 (56%) |
| Death Receptor Signaling | 2.12 | 15.1% | 1.069 | 3/93 (3%) | 0/93 (0%) | 11/93 (12%) | 79/93 (85%) |
| NAD biosynthesis II (from tryptophan) | 2.10 | 33.3% | -2.236 | 5/15 (33%) | 0/15 (0%) | 0/15 (0%) | 10/15 (67%) |
| Triacylglycerol Biosynthesis | 2.08 | 19.6% | -3 | 9/46 (20%) | 0/46 (0%) | 0/46 (0%) | 37/46 (80%) |
| Heparan Sulfate Biosynthesis (Late Stages) | 2.03 | 16.0% | -2.887 | 11/75 (15%) | 0/75 (0%) | 1/75 (1%) | 63/75 (84%) |
| Lysine Degradation V | 1.97 | 60.0% | N/A | 3/5 (60%) | 0/5 (0%) | 0/5 (0%) | 2/5 (40%) |
| Folate Polyglutamylation | 1.97 | 60.0% | N/A | 3/5 (60%) | 0/5 (0%) | 0/5 (0%) | 2/5 (40%) |
| Ketolysis | 1.96 | 40.0% | -2 | 4/10 (40%) | 0/10 (0%) | 0/10 (0%) | 6/10 (60%) |
| Neuroinflammation Signaling Pathway | 1.91 | 10.3% | 3.272 | 10/311 (3%) | 0/311 (0%) | 22/311 (7%) | 279/311 (90%) |
| Tumoricidal Function of Hepatic Natural Killer Cells | 1.88 | 25.0% | N/A | 2/24 (8%) | 0/24 (0%) | 4/24 (17%) | 18/24 (75%) |
| Mitochondrial L-carnitine Shuttle Pathway | 1.86 | 29.4% | -2.236 | 5/17 (29%) | 0/17 (0%) | 0/17 (0%) | 12/17 (71%) |
| Remodeling of Epithelial Adherens Junctions | 1.86 | 15.9% | 0 | 4/69 (6%) | 0/69 (0%) | 7/69 (10%) | 58/69 (84%) |
| TNFR1 Signaling | 1.85 | 18.0% | 1.414 | 2/50 (4%) | 0/50 (0%) | 7/50 (14%) | 41/50 (82%) |
| Apoptosis Signaling | 1.84 | 14.4% | 0.277 | 4/90 (4%) | 0/90 (0%) | 9/90 (10%) | 77/90 (86%) |
| AMPK Signaling | 1.84 | 11.1% | 0.728 | 16/216 (7%) | 0/216 (0%) | 8/216 (4%) | 192/216 (89%) |
| Mineralocorticoid Biosynthesis | 1.82 | 36.4% | -2 | 4/11 (36%) | 0/11 (0%) | 0/11 (0%) | 7/11 (64%) |
| NF-κB Signaling | 1.82 | 11.6% | 1.091 | 3/181 (2%) | 0/181 (0%) | 18/181 (10%) | 160/181 (88%) |
| Agranulocyte Adhesion and Diapedesis | 1.82 | 11.4% | N/A | 8/193 (4%) | 0/193 (0%) | 14/193 (7%) | 171/193 (89%) |
| Tryptophan Degradation X (Mammalian, via Tryptamine) | 1.82 | 24.0% | -2.449 | 6/25 (24%) | 0/25 (0%) | 0/25 (0%) | 19/25 (76%) |
| Caveolar-mediated Endocytosis Signaling | 1.79 | 15.5% | N/A | 5/71 (7%) | 0/71 (0%) | 6/71 (8%) | 60/71 (85%) |
| Epoxysqualene Biosynthesis | 1.78 | 100.0% | N/A | 2/2 (100%) | 0/2 (0%) | 0/2 (0%) | 0/2 (0%) |
| Glycine Biosynthesis III | 1.78 | 100.0% | N/A | 2/2 (100%) | 0/2 (0%) | 0/2 (0%) | 0/2 (0%) |
| Choline Degradation I | 1.78 | 100.0% | N/A | 2/2 (100%) | 0/2 (0%) | 0/2 (0%) | 0/2 (0%) |
| Cysteine Biosynthesis/Homocysteine Degradation | 1.78 | 100.0% | N/A | 2/2 (100%) | 0/2 (0%) | 0/2 (0%) | 0/2 (0%) |
| Glycine Biosynthesis I | 1.78 | 100.0% | N/A | 2/2 (100%) | 0/2 (0%) | 0/2 (0%) | 0/2 (0%) |
| Heparan Sulfate Biosynthesis | 1.78 | 14.6% | -2.887 | 11/82 (13%) | 0/82 (0%) | 1/82 (1%) | 70/82 (85%) |
| Arginine Biosynthesis IV | 1.76 | 50.0% | N/A | 3/6 (50%) | 0/6 (0%) | 0/6 (0%) | 3/6 (50%) |
| ILK Signaling | 1.74 | 11.2% | 1.789 | 8/197 (4%) | 0/197 (0%) | 14/197 (7%) | 175/197 (89%) |
| Atherosclerosis Signaling | 1.73 | 12.6% | N/A | 8/127 (6%) | 0/127 (0%) | 8/127 (6%) | 111/127 (87%) |
| TWEAK Signaling | 1.72 | 20.0% | -0.378 | 2/35 (6%) | 0/35 (0%) | 5/35 (14%) | 28/35 (80%) |
| IL-17A Signaling in Fibroblasts | 1.72 | 20.0% | N/A | 0/35 (0%) | 0/35 (0%) | 7/35 (20%) | 28/35 (80%) |
| Glucocorticoid Biosynthesis | 1.71 | 33.3% | -2 | 4/12 (33%) | 0/12 (0%) | 0/12 (0%) | 8/12 (67%) |
| Role of Macrophages, Fibroblasts and Endothelial Cells in Rheumatoid Arthritis | 1.69 | 9.9% | N/A | 8/313 (3%) | 0/313 (0%) | 23/313 (7%) | 282/313 (90%) |
| p38 MAPK Signaling | 1.60 | 12.5% | 2.84 | 1/120 (1%) | 0/120 (0%) | 14/120 (12%) | 105/120 (88%) |
| PPARα/RXRα Activation | 1.58 | 11.1% | -1.886 | 12/180 (7%) | 0/180 (0%) | 8/180 (4%) | 160/180 (89%) |
| Acetyl-CoA Biosynthesis I (Pyruvate Dehydrogenase Complex) | 1.57 | 42.9% | N/A | 3/7 (43%) | 0/7 (0%) | 0/7 (0%) | 4/7 (57%) |
| Aspartate Degradation II | 1.57 | 42.9% | N/A | 2/7 (29%) | 0/7 (0%) | 1/7 (14%) | 4/7 (57%) |
| Amyotrophic Lateral Sclerosis Signaling | 1.54 | 12.6% | 0.535 | 8/111 (7%) | 0/111 (0%) | 6/111 (5%) | 97/111 (87%) |
| Maturity Onset Diabetes of Young (MODY) Signaling | 1.53 | 23.8% | N/A | 5/21 (24%) | 0/21 (0%) | 0/21 (0%) | 16/21 (76%) |
| CD40 Signaling | 1.50 | 13.9% | 0.302 | 2/79 (3%) | 0/79 (0%) | 9/79 (11%) | 68/79 (86%) |
| Inhibition of Matrix Metalloproteases | 1.48 | 17.9% | 1.342 | 4/39 (10%) | 0/39 (0%) | 3/39 (8%) | 32/39 (82%) |
| Role of IL-17A in Arthritis | 1.48 | 14.5% | N/A | 2/69 (3%) | 0/69 (0%) | 8/69 (12%) | 59/69 (86%) |
| Polyamine Regulation in Colon Cancer | 1.45 | 22.7% | N/A | 3/22 (14%) | 0/22 (0%) | 2/22 (9%) | 17/22 (77%) |
| Fatty Acid α-oxidation | 1.45 | 22.7% | -2.236 | 5/22 (23%) | 0/22 (0%) | 0/22 (0%) | 17/22 (77%) |
| Osteoarthritis Pathway | 1.43 | 10.4% | 1.414 | 4/212 (2%) | 0/212 (0%) | 18/212 (8%) | 190/212 (90%) |
| L-carnitine Biosynthesis | 1.41 | 66.7% | N/A | 2/3 (67%) | 0/3 (0%) | 0/3 (0%) | 1/3 (33%) |
| Methylglyoxal Degradation I | 1.41 | 66.7% | N/A | 2/3 (67%) | 0/3 (0%) | 0/3 (0%) | 1/3 (33%) |
| Proline Degradation | 1.41 | 66.7% | N/A | 2/3 (67%) | 0/3 (0%) | 0/3 (0%) | 1/3 (33%) |
| Glutathione Biosynthesis | 1.41 | 66.7% | N/A | 2/3 (67%) | 0/3 (0%) | 0/3 (0%) | 1/3 (33%) |
| Tetrahydrobiopterin Biosynthesis I | 1.41 | 66.7% | N/A | 2/3 (67%) | 0/3 (0%) | 0/3 (0%) | 1/3 (33%) |
| Methionine Salvage II (Mammalian) | 1.41 | 66.7% | N/A | 2/3 (67%) | 0/3 (0%) | 0/3 (0%) | 1/3 (33%) |
| 4-hydroxyproline Degradation I | 1.41 | 66.7% | N/A | 2/3 (67%) | 0/3 (0%) | 0/3 (0%) | 1/3 (33%) |
| Tetrahydrobiopterin Biosynthesis II | 1.41 | 66.7% | N/A | 2/3 (67%) | 0/3 (0%) | 0/3 (0%) | 1/3 (33%) |
| Glutamate Degradation II | 1.41 | 66.7% | N/A | 1/3 (33%) | 0/3 (0%) | 1/3 (33%) | 1/3 (33%) |
| Tyrosine Biosynthesis IV | 1.41 | 66.7% | N/A | 2/3 (67%) | 0/3 (0%) | 0/3 (0%) | 1/3 (33%) |
| L-serine Degradation | 1.41 | 66.7% | N/A | 0/3 (0%) | 0/3 (0%) | 2/3 (67%) | 1/3 (33%) |
| Aspartate Biosynthesis | 1.41 | 66.7% | N/A | 1/3 (33%) | 0/3 (0%) | 1/3 (33%) | 1/3 (33%) |
| Glycogen Degradation III | 1.41 | 26.7% | -2 | 4/15 (27%) | 0/15 (0%) | 0/15 (0%) | 11/15 (73%) |
| Activation of IRF by Cytosolic Pattern Recognition Receptors | 1.33 | 14.3% | 0.333 | 1/63 (2%) | 0/63 (0%) | 8/63 (13%) | 54/63 (86%) |
| IL-17 Signaling | 1.33 | 12.9% | N/A | 2/85 (2%) | 0/85 (0%) | 9/85 (11%) | 74/85 (87%) |
| Sucrose Degradation V (Mammalian) | 1.31 | 33.3% | N/A | 3/9 (33%) | 0/9 (0%) | 0/9 (0%) | 6/9 (67%) |
| Phosphatidylethanolamine Biosynthesis II | 1.31 | 33.3% | N/A | 3/9 (33%) | 0/9 (0%) | 0/9 (0%) | 6/9 (67%) |
| Leucine Degradation I | 1.31 | 33.3% | N/A | 3/9 (33%) | 0/9 (0%) | 0/9 (0%) | 6/9 (67%) |
| Folate Transformations I | 1.31 | 33.3% | N/A | 3/9 (33%) | 0/9 (0%) | 0/9 (0%) | 6/9 (67%) |
| Hypoxia Signaling in the Cardiovascular System | 1.30 | 13.3% | 2.646 | 2/75 (3%) | 0/75 (0%) | 8/75 (11%) | 65/75 (87%) |
