# Supplementary material for: Cyclophilin D‐dependent mitochondrial permeability transition amplifies inflammatory reprogramming in endotoxemia
Source: FEBS Open Bio. 2021 Feb 13;11(3):684–704. doi: 10.1002/2211-5463.13091 (PMC7931201; doi:10.1002/2211-5463.13091)
Supplement: Supplementary file 6 — Table S3. List of DEGs in the CypD‐/‐+LPS vs WT+LPS comparison in canonical pathways analyzed by IPA. ‐lg(adjPval.) > 1.3. [file FEB4-11-684-s006.docx]

| **Ingenuity Canonical Pathways** | **-log(B-H p-value)** | **Ratio** | **z-score** | **Downregulated** | **No change** | **Upregulated** | **No overlap with dataset** |
| --- | --- | --- | --- | --- | --- | --- | --- |
| LPS/IL-1 Mediated Inhibition of RXR Function | 14.80 | 19.4% | -2.357 | 11/222 (5%) | 0/222 (0%) | 32/222 (14%) | 179/222 (81%) |
| Mitochondrial Dysfunction | 12.70 | 20.5% | N/A | 1/171 (1%) | 0/171 (0%) | 34/171 (20%) | 136/171 (80%) |
| Superpathway of Cholesterol Biosynthesis | 11.50 | 53.6% | 3.873 | 0/28 (0%) | 0/28 (0%) | 15/28 (54%) | 13/28 (46%) |
| Sirtuin Signaling Pathway | 10.30 | 14.4% | -1.715 | 14/292 (5%) | 0/292 (0%) | 28/292 (10%) | 250/292 (86%) |
| FXR/RXR Activation | 10.30 | 21.4% | N/A | 8/126 (6%) | 0/126 (0%) | 19/126 (15%) | 99/126 (79%) |
| Cholesterol Biosynthesis I | 9.82 | 76.9% | 3.162 | 0/13 (0%) | 0/13 (0%) | 10/13 (77%) | 3/13 (23%) |
| Cholesterol Biosynthesis II (via 24,25-dihydrolanosterol) | 9.82 | 76.9% | 3.162 | 0/13 (0%) | 0/13 (0%) | 10/13 (77%) | 3/13 (23%) |
| Cholesterol Biosynthesis III (via Desmosterol) | 9.82 | 76.9% | 3.162 | 0/13 (0%) | 0/13 (0%) | 10/13 (77%) | 3/13 (23%) |
| Oxidative Phosphorylation | 9.52 | 22.0% | 4.899 | 0/109 (0%) | 0/109 (0%) | 24/109 (22%) | 85/109 (78%) |
| LXR/RXR Activation | 9.36 | 20.7% | 2.236 | 11/121 (9%) | 0/121 (0%) | 14/121 (12%) | 96/121 (79%) |
| PXR/RXR Activation | 8.72 | 27.7% | N/A | 6/65 (9%) | 0/65 (0%) | 12/65 (18%) | 47/65 (72%) |
| Fatty Acid β-oxidation I | 8.40 | 40.6% | 3.051 | 1/32 (3%) | 0/32 (0%) | 12/32 (38%) | 19/32 (59%) |
| Nicotine Degradation II | 7.82 | 26.2% | 2.668 | 3/65 (5%) | 0/65 (0%) | 14/65 (22%) | 48/65 (74%) |
| Acetone Degradation I (to Methylglyoxal) | 7.64 | 40.0% | 1.732 | 3/30 (10%) | 0/30 (0%) | 9/30 (30%) | 18/30 (60%) |
| Nicotine Degradation III | 7.00 | 26.8% | 2.324 | 3/56 (5%) | 0/56 (0%) | 12/56 (21%) | 41/56 (73%) |
| Melatonin Degradation I | 6.97 | 24.6% | 1.5 | 5/65 (8%) | 0/65 (0%) | 11/65 (17%) | 49/65 (75%) |
| Xenobiotic Metabolism Signaling | 6.85 | 12.1% | N/A | 10/290 (3%) | 0/290 (0%) | 25/290 (9%) | 255/290 (88%) |
| Ethanol Degradation II | 6.54 | 32.4% | 3.464 | 0/37 (0%) | 0/37 (0%) | 12/37 (32%) | 25/37 (68%) |
| Superpathway of Melatonin Degradation | 6.54 | 22.9% | 1.5 | 5/70 (7%) | 0/70 (0%) | 11/70 (16%) | 54/70 (77%) |
| Glycine Betaine Degradation | 6.37 | 70.0% | 2.646 | 0/10 (0%) | 0/10 (0%) | 7/10 (70%) | 3/10 (30%) |
| Bupropion Degradation | 6.37 | 40.0% | 1.265 | 3/25 (12%) | 0/25 (0%) | 7/25 (28%) | 15/25 (60%) |
| Estrogen Biosynthesis | 6.05 | 29.3% | 1.732 | 3/41 (7%) | 0/41 (0%) | 9/41 (22%) | 29/41 (71%) |
| Serotonin Degradation | 6.00 | 20.8% | 3 | 2/77 (3%) | 0/77 (0%) | 14/77 (18%) | 61/77 (79%) |
| Hepatic Cholestasis | 5.73 | 14.4% | N/A | 9/160 (6%) | 0/160 (0%) | 14/160 (9%) | 137/160 (86%) |
| Bile Acid Biosynthesis, Neutral Pathway | 5.36 | 53.8% | 2.646 | 0/13 (0%) | 0/13 (0%) | 7/13 (54%) | 6/13 (46%) |
| Ethanol Degradation IV | 5.27 | 36.0% | 3 | 0/25 (0%) | 0/25 (0%) | 9/25 (36%) | 16/25 (64%) |
| Histidine Degradation VI | 4.85 | 46.7% | 2.646 | 0/15 (0%) | 0/15 (0%) | 7/15 (47%) | 8/15 (53%) |
| Oxidative Ethanol Degradation III | 4.85 | 38.1% | 2.828 | 0/21 (0%) | 0/21 (0%) | 8/21 (38%) | 13/21 (62%) |
| Acute Phase Response Signaling | 4.73 | 12.9% | -1.414 | 13/170 (8%) | 0/170 (0%) | 9/170 (5%) | 148/170 (87%) |
| Noradrenaline and Adrenaline Degradation | 4.34 | 25.0% | 3.162 | 0/40 (0%) | 0/40 (0%) | 10/40 (25%) | 30/40 (75%) |
| Tryptophan Degradation III (Eukaryotic) | 4.23 | 32.0% | 2.828 | 0/25 (0%) | 0/25 (0%) | 8/25 (32%) | 17/25 (68%) |
| Histidine Degradation III | 4.09 | 62.5% | 2.236 | 0/8 (0%) | 0/8 (0%) | 5/8 (63%) | 3/8 (38%) |
| Iron homeostasis signaling pathway | 4.09 | 13.5% | N/A | 6/133 (5%) | 0/133 (0%) | 12/133 (9%) | 115/133 (86%) |
| Tyrosine Degradation I | 3.74 | 80.0% | 1 | 1/5 (20%) | 0/5 (0%) | 3/5 (60%) | 1/5 (20%) |
| Folate Polyglutamylation | 3.74 | 80.0% | 2 | 0/5 (0%) | 0/5 (0%) | 4/5 (80%) | 1/5 (20%) |
| Glutathione-mediated Detoxification | 3.52 | 25.8% | 2.828 | 0/31 (0%) | 0/31 (0%) | 8/31 (26%) | 23/31 (74%) |
| Glutathione Redox Reactions I | 3.39 | 29.2% | 2.646 | 0/24 (0%) | 0/24 (0%) | 7/24 (29%) | 17/24 (71%) |
| Glycine Cleavage Complex | 3.31 | 66.7% | 1 | 1/6 (17%) | 0/6 (0%) | 3/6 (50%) | 2/6 (33%) |
| Tryptophan Degradation X (Mammalian, via Tryptamine) | 3.28 | 28.0% | 2.646 | 0/25 (0%) | 0/25 (0%) | 7/25 (28%) | 18/25 (72%) |
| Aryl Hydrocarbon Receptor Signaling | 3.25 | 12.1% | 0.447 | 5/141 (4%) | 0/141 (0%) | 12/141 (9%) | 124/141 (88%) |
| Valine Degradation I | 3.22 | 33.3% | 1.633 | 1/18 (6%) | 0/18 (0%) | 5/18 (28%) | 12/18 (67%) |
| Histamine Degradation | 3.08 | 31.6% | 2.449 | 0/19 (0%) | 0/19 (0%) | 6/19 (32%) | 13/19 (68%) |
| EIF2 Signaling | 3.07 | 10.0% | 0.832 | 13/221 (6%) | 0/221 (0%) | 9/221 (4%) | 199/221 (90%) |
| Dopamine Degradation | 3.01 | 21.6% | 1.414 | 2/37 (5%) | 0/37 (0%) | 6/37 (16%) | 29/37 (78%) |
| Isoleucine Degradation I | 2.78 | 35.7% | 1.342 | 1/14 (7%) | 0/14 (0%) | 4/14 (29%) | 9/14 (64%) |
| Androgen Biosynthesis | 2.78 | 35.7% | 2.236 | 0/14 (0%) | 0/14 (0%) | 5/14 (36%) | 9/14 (64%) |
| Tryptophan Degradation to 2-amino-3-carboxymuconate Semialdehyde | 2.76 | 50.0% | 2 | 0/8 (0%) | 0/8 (0%) | 4/8 (50%) | 4/8 (50%) |
| Fatty Acid α-oxidation | 2.74 | 27.3% | 2.449 | 0/22 (0%) | 0/22 (0%) | 6/22 (27%) | 16/22 (73%) |
| NAD biosynthesis II (from tryptophan) | 2.64 | 33.3% | 2.236 | 0/15 (0%) | 0/15 (0%) | 5/15 (33%) | 10/15 (67%) |
| Putrescine Degradation III | 2.64 | 26.1% | 2.449 | 0/23 (0%) | 0/23 (0%) | 6/23 (26%) | 17/23 (74%) |
| Phenylalanine Degradation I (Aerobic) | 2.59 | 75.0% | N/A | 0/4 (0%) | 0/4 (0%) | 3/4 (75%) | 1/4 (25%) |
| Heme Biosynthesis II | 2.58 | 44.4% | 2 | 0/9 (0%) | 0/9 (0%) | 4/9 (44%) | 5/9 (56%) |
| Folate Transformations I | 2.58 | 44.4% | 2 | 0/9 (0%) | 0/9 (0%) | 4/9 (44%) | 5/9 (56%) |
| TCA Cycle II (Eukaryotic) | 2.57 | 25.0% | 1.633 | 1/24 (4%) | 0/24 (0%) | 5/24 (21%) | 18/24 (75%) |
| Unfolded protein response | 2.57 | 16.4% | N/A | 6/55 (11%) | 0/55 (0%) | 3/55 (5%) | 46/55 (84%) |
| PPARα/RXRα Activation | 2.57 | 10.0% | 0.728 | 13/180 (7%) | 0/180 (0%) | 5/180 (3%) | 162/180 (90%) |
| Stearate Biosynthesis I (Animals) | 2.57 | 18.2% | 2.121 | 1/44 (2%) | 0/44 (0%) | 7/44 (16%) | 36/44 (82%) |
| Superpathway of Geranylgeranyldiphosphate Biosynthesis I (via Mevalonate) | 2.43 | 29.4% | 2.236 | 0/17 (0%) | 0/17 (0%) | 5/17 (29%) | 12/17 (71%) |
| Glycolysis I | 2.41 | 23.1% | 2.449 | 0/26 (0%) | 0/26 (0%) | 6/26 (23%) | 20/26 (77%) |
| Gluconeogenesis I | 2.41 | 23.1% | 2.449 | 0/26 (0%) | 0/26 (0%) | 6/26 (23%) | 20/26 (77%) |
| Superpathway of Methionine Degradation | 2.33 | 18.9% | 1.134 | 2/37 (5%) | 0/37 (0%) | 5/37 (14%) | 30/37 (81%) |
| Tetrapyrrole Biosynthesis II | 2.30 | 60.0% | N/A | 0/5 (0%) | 0/5 (0%) | 3/5 (60%) | 2/5 (40%) |
| Lysine Degradation V | 2.30 | 60.0% | N/A | 0/5 (0%) | 0/5 (0%) | 3/5 (60%) | 2/5 (40%) |
| Mineralocorticoid Biosynthesis | 2.27 | 36.4% | 2 | 0/11 (0%) | 0/11 (0%) | 4/11 (36%) | 7/11 (64%) |
| Ubiquinol-10 Biosynthesis (Eukaryotic) | 2.24 | 26.3% | 2.236 | 0/19 (0%) | 0/19 (0%) | 5/19 (26%) | 14/19 (74%) |
| Methylglyoxal Degradation III | 2.24 | 26.3% | 2.236 | 0/19 (0%) | 0/19 (0%) | 5/19 (26%) | 14/19 (74%) |
| Toll-like Receptor Signaling | 2.20 | 13.2% | -2.646 | 10/76 (13%) | 0/76 (0%) | 0/76 (0%) | 66/76 (87%) |
| Pregnenolone Biosynthesis | 2.14 | 33.3% | 2 | 0/12 (0%) | 0/12 (0%) | 4/12 (33%) | 8/12 (67%) |
| Glucocorticoid Biosynthesis | 2.14 | 33.3% | 2 | 0/12 (0%) | 0/12 (0%) | 4/12 (33%) | 8/12 (67%) |
| Zymosterol Biosynthesis | 2.06 | 50.0% | N/A | 0/6 (0%) | 0/6 (0%) | 3/6 (50%) | 3/6 (50%) |
| Maturity Onset Diabetes of Young (MODY) Signaling | 2.06 | 23.8% | N/A | 0/21 (0%) | 0/21 (0%) | 5/21 (24%) | 16/21 (76%) |
| Mevalonate Pathway I | 2.01 | 30.8% | 2 | 0/13 (0%) | 0/13 (0%) | 4/13 (31%) | 9/13 (69%) |
| Thyroid Hormone Metabolism II (via Conjugation and/or Degradation) | 2.00 | 16.3% | 1.134 | 2/43 (5%) | 0/43 (0%) | 5/43 (12%) | 36/43 (84%) |
| IL-10 Signaling | 1.97 | 13.0% | N/A | 8/69 (12%) | 0/69 (0%) | 1/69 (1%) | 60/69 (87%) |
| Epoxysqualene Biosynthesis | 1.97 | 100.0% | N/A | 0/2 (0%) | 0/2 (0%) | 2/2 (100%) | 0/2 (0%) |
| Choline Degradation I | 1.97 | 100.0% | N/A | 0/2 (0%) | 0/2 (0%) | 2/2 (100%) | 0/2 (0%) |
| Cysteine Biosynthesis/Homocysteine Degradation | 1.97 | 100.0% | N/A | 0/2 (0%) | 0/2 (0%) | 2/2 (100%) | 0/2 (0%) |
| Adenine and Adenosine Salvage I | 1.97 | 100.0% | N/A | 1/2 (50%) | 0/2 (0%) | 1/2 (50%) | 0/2 (0%) |
| Glycine Biosynthesis I | 1.97 | 100.0% | N/A | 0/2 (0%) | 0/2 (0%) | 2/2 (100%) | 0/2 (0%) |
| TR/RXR Activation | 1.95 | 11.2% | N/A | 4/98 (4%) | 0/98 (0%) | 7/98 (7%) | 87/98 (89%) |
| NRF2-mediated Oxidative Stress Response | 1.94 | 8.8% | 1.633 | 6/193 (3%) | 0/193 (0%) | 11/193 (6%) | 176/193 (91%) |
| Retinoate Biosynthesis I | 1.89 | 17.6% | 2.449 | 0/34 (0%) | 0/34 (0%) | 6/34 (18%) | 28/34 (82%) |
| Glutaryl-CoA Degradation | 1.71 | 25.0% | 2 | 0/16 (0%) | 0/16 (0%) | 4/16 (25%) | 12/16 (75%) |
| Activation of IRF by Cytosolic Pattern Recognition Receptors | 1.68 | 12.7% | -0.707 | 7/63 (11%) | 0/63 (0%) | 1/63 (2%) | 55/63 (87%) |
| γ-linolenate Biosynthesis II (Animals) | 1.61 | 23.5% | 2 | 0/17 (0%) | 0/17 (0%) | 4/17 (24%) | 13/17 (76%) |
| Leucine Degradation I | 1.57 | 33.3% | N/A | 0/9 (0%) | 0/9 (0%) | 3/9 (33%) | 6/9 (67%) |
| Production of Nitric Oxide and Reactive Oxygen Species in Macrophages | 1.57 | 8.3% | -1.291 | 8/194 (4%) | 0/194 (0%) | 8/194 (4%) | 178/194 (92%) |
| Atherosclerosis Signaling | 1.57 | 9.5% | N/A | 7/127 (6%) | 0/127 (0%) | 5/127 (4%) | 115/127 (91%) |
| Methylglyoxal Degradation I | 1.57 | 66.7% | N/A | 0/3 (0%) | 0/3 (0%) | 2/3 (67%) | 1/3 (33%) |
| Methionine Salvage II (Mammalian) | 1.57 | 66.7% | N/A | 0/3 (0%) | 0/3 (0%) | 2/3 (67%) | 1/3 (33%) |
| Thiosulfate Disproportionation III (Rhodanese) | 1.57 | 66.7% | N/A | 0/3 (0%) | 0/3 (0%) | 2/3 (67%) | 1/3 (33%) |
| Tyrosine Biosynthesis IV | 1.57 | 66.7% | N/A | 0/3 (0%) | 0/3 (0%) | 2/3 (67%) | 1/3 (33%) |
| Prostate Cancer Signaling | 1.54 | 10.3% | N/A | 8/97 (8%) | 0/97 (0%) | 2/97 (2%) | 87/97 (90%) |
| Ketogenesis | 1.46 | 30.0% | N/A | 0/10 (0%) | 0/10 (0%) | 3/10 (30%) | 7/10 (70%) |
| p38 MAPK Signaling | 1.34 | 9.2% | -3.317 | 11/120 (9%) | 0/120 (0%) | 0/120 (0%) | 109/120 (91%) |
| iNOS Signaling | 1.34 | 13.3% | -2.449 | 6/45 (13%) | 0/45 (0%) | 0/45 (0%) | 39/45 (87%) |
| Endoplasmic Reticulum Stress Pathway | 1.33 | 19.0% | N/A | 4/21 (19%) | 0/21 (0%) | 0/21 (0%) | 17/21 (81%) |
| Uracil Degradation II (Reductive) | 1.33 | 50.0% | N/A | 0/4 (0%) | 0/4 (0%) | 2/4 (50%) | 2/4 (50%) |
| Branched-chain α-keto acid Dehydrogenase Complex | 1.33 | 50.0% | N/A | 1/4 (25%) | 0/4 (0%) | 1/4 (25%) | 2/4 (50%) |
| α-tocopherol Degradation | 1.33 | 50.0% | N/A | 0/4 (0%) | 0/4 (0%) | 2/4 (50%) | 2/4 (50%) |
| Glutathione Redox Reactions II | 1.33 | 50.0% | N/A | 2/4 (50%) | 0/4 (0%) | 0/4 (0%) | 2/4 (50%) |
| Thymine Degradation | 1.33 | 50.0% | N/A | 0/4 (0%) | 0/4 (0%) | 2/4 (50%) | 2/4 (50%) |
| Acetate Conversion to Acetyl-CoA | 1.33 | 50.0% | N/A | 0/4 (0%) | 0/4 (0%) | 2/4 (50%) | 2/4 (50%) |
| Fatty Acid β-oxidation III (Unsaturated, Odd Number) | 1.33 | 50.0% | N/A | 1/4 (25%) | 0/4 (0%) | 1/4 (25%) | 2/4 (50%) |
